# A novel set of volatile urinary biomarkers for late-life major depressive and anxiety disorders upon the progression of frailty: a pilot study

**DOI:** 10.1007/s44192-022-00023-0

**Published:** 2022-10-27

**Authors:** Akiko Fujita, Kazushige Ihara, Hisashi Kawai, Shuichi Obuchi, Yutaka Watanabe, Hirohiko Hirano, Yoshinori Fujiwara, Yoichi Takeda, Masashi Tanaka, Keiko Kato

**Affiliations:** 1 Faculty of Life Sciences, Kyoto Sangyo University, Motoyama, Kamigamo, Kita-Ku, Kyoto, 603-8555 Japan; 2 Department of Social Medicine, Graduate School of Medicine and School of Medicine, Hirosaki University, 5 Zaifu-Cho Hirosaki City, Aomori, 036-8562 Japan; 3 Research Team for Human Care, Tokyo Metropolitan Institute of Gerontology, 35-2 Sakae-Cho, Itabashi-Ku, Tokyo, 173-0015 Japan; 4 Gerodontology, Department of Oral Health Science, Faculty of Dental Medicine, Hokkaido University, Kita13, Nishi7, Kita-Ku, Sapporo, Hokkaido, 060-8586 Japan; 5 Research Team for Promoting Independence and Mental Health, Tokyo Metropolitan Institute of Gerontology, 35-2 Sakae-Cho, Itabashi-Ku, Tokyo, 173-0015 Japan; 6 Research Team for Social Participation and Community Health, Tokyo Metropolitan Institute of Gerontology, 35-2 Sakae-Cho, Itabashi-Ku, Tokyo, 173-0015 Japan; 7 Department of Biotechnology, College of Life Sciences, Ritsumeikan University, 1-1-1 Noji-Higashi, Kusatsu, Shiga 525-8577 Japan; 8 Department of Neurology, Juntendo University Graduate School of Medicine, 2-1-1 Hongo, Bunkyo-Ku, Tokyo, 113-8421 Japan

**Keywords:** Biomarker, Depression, Anxiety/agoraphobia, Frailty, Elderly people, Non-invasive, Urine, Volatile organic compounds (VOCs)

## Abstract

Mood and anxiety disorders are frequent in the elderly and increase the risk of frailty. This study aimed to identify novel biomarkers of major depressive disorder (MDD) and anxiety in the elderly. We examined 639 participants in the community-dwelling Otassha Study (518 individuals considered healthy control, 77 with depression, anxiety, etc.), mean age 75 years, 58.4% of female. After exclusion criteria, we analyzed VOCs from 18 individuals (9 healthy control, 9 of MDD/agoraphobia case). Urinary volatile and semi-volatile organic compounds (VOCs) were profiled using solid-phase microextraction and gas chromatography-mass spectrometry. Six urinary VOCs differed in the absolute area of the base peak between participants with MDD and/or agoraphobia and controls. High area under the receiver-operating characteristic curve (AUC) values were found for phenethyl isothiocyanate (AUC: 0.86, *p* = 0.009), hexanoic acid (AUC: 0.85, *p* = 0.012), texanol (AUC: 0.99, *p* = 0.0005), and texanol isomer (AUC: 0.89, *p* = 0.005). The combined indices of dimethyl sulfone, phenethyl isothiocyanate, and hexanoic acid, and texanol and texanol isomer showed AUCs of 0.91 (*p* = 0.003) and 0.99 (*p* = 0.0005) and correlated with the GRID-HAMD and the Kihon Checklist (CL score), respectively. These VOCs may be valuable biomarkers for evaluating MDD and/or agoraphobia in the elderly.

**Supplementary Information:**

The online version contains supplementary material available at 10.1007/s44192-022-00023-0.

## Introduction

Epidemiological analyses in the elderly have indicated that MDD reaches its highest incidence in late life, with a prevalence of 1–4% in community-dwelling and 10–12% in hospitalized elderly [1–3], thereby decreasing quality of life, and is strongly correlated with the progression of frailty [2, 4]. Anxiety disorders are estimated to have a prevalence of 1.2–15% in community-dwelling elderly, with agoraphobia being the most frequent [3, 5]. Frailty is a syndrome wherein the accumulation of age-related decline in several physiological functions results in increased vulnerability to stress and a tendency for homeostasis disruption [6, 7]. The progression of frailty can lead to delirium, the need for nursing care, and institutionalization. Prospective studies of the relationship between frailty and MDD and anxiety suggest that depression and anxiety are associated with increased weakness, mobility deficits, and fatigue, increasing the risk of frailty and mortality over a period of up to 5 years [8, 9]; however, medications are associated with a higher risk of frailty [10–12]. Therefore, non-pharmacological interventions and early detection are essential for managing frailty and lowering the risks of depression and anxiety.

Screening for depression, anxiety, and frailty have mainly been based on the subjective identification of symptoms, using comprehensive questionnaires assessing depression [1, 2, 13], anxiety [3], and frailty [14, 15]. The diagnosis of depression and anxiety requires knowledge of the mental phenomena and psychological events of the patient, while that of frailty including depression and anxiety requires a full understanding of the patient’s physiological, psychological, functional, and social status. For instance, for a concrete clinical diagnosis, a psychiatrist can identify persons with MDD using the mood episodes (A) module in the Structured Clinical Interview for the Diagnostic and Statistical Manual of Mental Disorders, fourth edition (DSM-IV) (SCID) [16, 17], and assess the severity of MDD symptoms on the GRID-Hamilton Rating Scale for Depression (HAMD) [13]. Types of anxiety disorders can be diagnosed according to the DSM-IV using the E, F, and G modules of the Mini-International Neuropsychiatric Interview (M.I.N.I.) [18, 19]. Furthermore, the psychiatrist may exclude psychiatric disorders other than MDD and anxiety (e.g., dysthymic disorder, alcoholism, schizophrenia, and dementia) using the DSM-IV [16]; thus, the clinical diagnosis of MDD and anxiety can be made with confidence. Generally, due to the heterogeneity of the disorder, diagnosis is difficult and time-consuming and requires considerable skill. Using early results of an objective diagnostic biochemical test with the lowest possible rate of false negatives in the elderly and under technical facilities with higher sensitivity and stability, psychiatrists can focus on the diagnosis and treatment of people with false-positive and positive results.

The search for biomarkers for depression and anxiety is focused on neuroimaging and molecules in the blood, saliva, urine, hair, feces, and cerebrospinal fluid (CSF) [20–24]. To date, neuroimaging and biological molecules including neurotrophic factors, neurotransmitters, immunological factors, hormones, and metabolites have been nominated as biomarkers. In the elderly, several studies have also indicated possible candidates of biomarkers specific to late-life depression and found that the levels of certain biomarkers, such as tau, Aβ, and Apo E, were different from those in general populations [25]. Generally, however, no established biomarkers show prospective evidence for the onset/relapse/recurrence of depression used in clinical practice to identify individuals with MDD [23]. Neuro-imaging studies suggest that anxiety among the elderly can be a prodromal marker of Alzheimer’s disease and vascular dementia [5].

Odors emanating from the human body have long been used to detect signs of certain diseases [26, 27]. Odors consist of mixtures of many volatile organic compounds (VOCs) emitted from breath, urine, sweat, and feces, which are the end products of the body's metabolic system. Therefore, theoretically, the pathological processes of endogenous metabolic disorders can influence the composition of VOCs within odors, and the production of VOCs would change depending on the frailty status and physiological functions in the limbic system of the brain; thus, symptoms of depression and anxiety would affect the VOC composition. No prior studies have used VOCs to evaluate psychiatric conditions; however, we obtained a Japanese patent (No. JPA2019-82367, Patented before issuing the patent gazette) for the six VOCs found to be significant in this study as “Depression and anxiety biomarkers in human urine.”

Previously, we used gas chromatography-mass spectrometry (GC-MS) to detect VOCs obtained by heating mouse urine at 45 °C for 1 h. Urinary biomarkers of temporal lobe epilepsy (TLE) were identified in amygdala-kindling mice [28] and for depression and anxiety in sialyltransferase St3gal4-deficient mice [29]. The urinary metabolites of epilepsy and depressive anxiety included differential VOCs specific to each disease. Many urinary VOCs belonged to hydrocarbons and derivatives of fatty acids groups, which are metabolized by the mevalonate pathway [28, 29]. The mevalonate pathway also metabolizes cholesterol, which is composed of sex hormones, suggesting the possibility that sex dimorphism and age affect the detection of VOC biomarkers. However, the elderly may be less susceptible to the effects of sex hormones.

In this study, we first hypothesized that altered urinary VOC profiles could be derived from specific metabolic cascade alterations in the elderly with MDD and/or agoraphobia (an anxiety disorder). If so, the urinary VOC biomarkers could help identify key molecules involved in the specific metabolic cascades. These findings could lead to the development of novel medications and represent a non-invasive tool for primary screening for MDD/agoraphobia and frailty among the elderly.

## Methods

### Study population

This was a case–control study in a cohort for which urinary examination results were available. The Otassha Study 2011 cohort enrolled community-dwelling elderly, and a notification letter was mailed in 2011 to all 6699 residents aged 65–84 years living in the nine areas of Itabashi City, Tokyo. This baseline survey included 913 individuals [30]. The 2015 survey invited past participants (639 participants, 374 women, and 266 men) aged 66–88 years who underwent a comprehensive health survey between October 1 and 7, 2015. The survey, which included physical and psychological examinations and questionnaires, assessed the frailty and depressive symptoms of the subjects using the Kihon checklist score (CL score) [14], Tokyo Metropolitan Institute of Gerontology Index of Competence (TMIG-IC) [31], Japan Science and Technology Agency Index of Competence (JST-IC) [15], depression section of the Kihon Checklist (DSKC) [32], and Zung Self-Rating Depression Scale (SDS) [33]. We sequentially collected urine samples of 636 people between 11:30 and 17:30 h, regardless of whether it was before or after a meal. All participants provided informed consent. The study was approved by the Ethical Committees of the Tokyo Metropolitan Institute of Gerontology (approval number 2011H48), Kyoto Sangyo University (approval number 0026), and Toho University (approval number A17014).

### Assessment of frailty and MDD/agoraphobia

The Kihon checklist is a standard self-report questionnaire used to assess the progression of frailty [34]. It consists of 25 items including lifestyle, physical strength, nutrition and eating, and depression subdomains. The depression subdomains consist of five items that participants answer either “Yes” or “No” to, and one point is added when the answer corresponds to the factors known to increase the risk of depression. The present Kihon CL score in the Kihon checklist excluded the five questions in the depression subdomain, as described previously [14]. The depression subdomain in the Kihon checklist was analyzed as DSKC using self-report and report-by-others assessment methods [32]. The TMIG-IC [31] is a questionnaire assessing the progression of frailty and is accepted worldwide. It consists of 13 items related to self-reliance, intelligence, and social participation, and does not include questions concerning physical independence. Participants answer “Yes” or “No.” Scores of < 10 indicate a decrease in life function. The JST-IC [15] is another report-by-others questionnaire for assessing the progression of frailty and is a modified version of the TMIG-IC containing items adapted to the changes in the current living environment and lifestyle. It consists of 16 items concerning the subdomains of use of new devices, intelligence, life management, and social participation, each containing four items. Participants also answer “Yes” or “No.” The Zung SDS [33] is a 20-item questionnaire, with each item rated on a 4-point scale. Participants responded by answering from rank 1 to 4, with each response receiving a point; this might be a “rank 1” to a negative question or a positive question. Scores are generally classified as normal (< 50), mild depression (50–59), moderate to marked major depression (60–69), and severe to extreme major depression (> 70) [35]. The SDS was previously translated and validated in the Japanese population, fortifying the reliability of key study methods [36]. Additionally, persons who scored ≤ 23 on the Mini-Mental State Examination (MMSE) [37] were excluded due to suspected cognitive deficits.

**Fig. 1:**
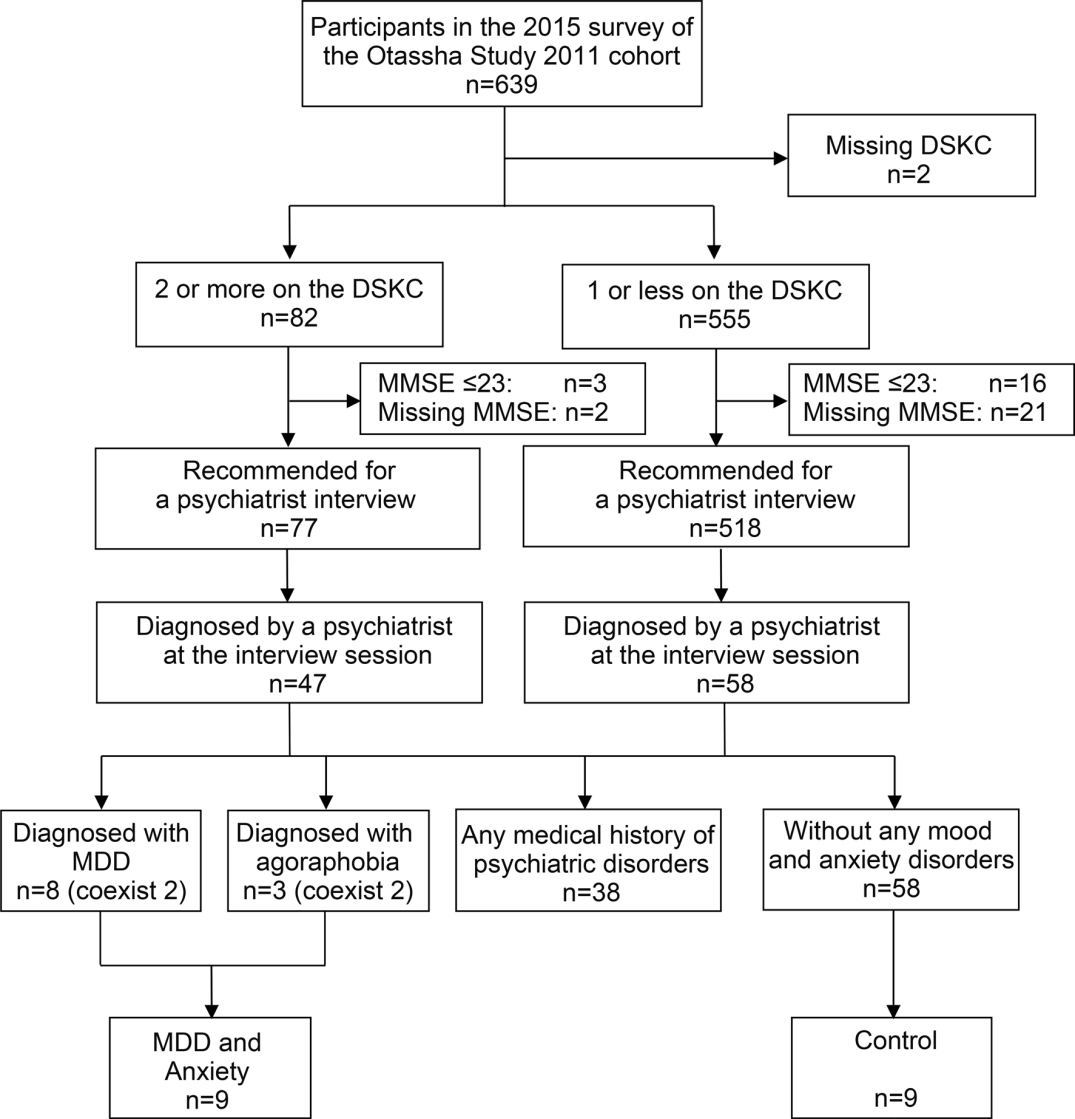
Flowchart for identifying persons suffering from major depressive disorder (MDD) and/or agoraphobia disorders *DSKC* the depression section of the Kihon Checklist, *MMSE* the Mini-Mental State Examination

The occurrence of mood (major depressive disorder [MDD], dysthymia, and bipolar disorder), anxiety (agoraphobia, panic disorder, phobia, generalized anxiety disorder, and post-traumatic stress disorder), and coexisting mood-anxiety disorders has been assessed using criteria prescribed in the DSM-IV [16]. MDD is an important symptom as it is both a cause and result of the progression of frailty and is often accompanied by anxiety as a comorbidity [2–5]. The psychiatrist identified persons suffering from anxiety among participants with possible mood disorders extracted using the DSKC self-score as a screening tool. We then investigated urinary VOCs in persons with MDD (a mood disorder) and/or agoraphobia (an anxiety disorder). Figure 1 shows the groups and the numbers in each step for identifying persons suffering from MDD and/or agoraphobia and the negative controls. Of 639 participants aged 66–88 years in The Otassha Study 2011 cohort 2015 survey, 77 persons scored ≥ 24 on the MMSE and ≥ 2 on the DSKC using report-by-others assessment methods [32] and were suspected to have depression as “Recommended for a psychiatric interview.” Additionally, 518 persons who scored ≥ 24 on the MMSE and with ≤ 1 on the DSKC using report-by-others assessment methods were negative candidates for MDD and anxiety as “Recommended for a psychiatric interview.” A total of 10–15 negative candidates were selected randomly per day for 7 days among 518 persons (recommended for a psychiatric interview), and their willingness was considered. The willingness of 77 positive candidates (recommended for a psychiatric interview) for the interview was noted. Subsequently, 47 positive and 58 negative candidates agreed to a psychiatric interview. The psychiatrist determined whether these candidates suffered from depression through the interview sessions using the mood episodes (A) module of the SCID [17] and identified eight persons who fulfilled the criteria for MDD on the SCID and scored ≥ 7 on the GRID-HAMD [13]. The psychiatrist also identified three persons with agoraphobia according to the DSM-IV using the E, F, and G modules of the M.I.N.I. [18, 19]. The three modules provided the psychiatrists with tools to identify subtypes of anxiety disorders, such as panic disorder, agoraphobia, and social phobia; the psychiatrist then determined whether the person experienced anxiety. Finally, nine individuals with MDD and/or agoraphobia were identified (Fig. 1). That is, six persons had MDD, two experienced both MDD and agoraphobia and one had agoraphobia without MDD. None were determined to have anxiety aside from agoraphobia among those diagnosed by the psychiatrist. Among the 58 persons without any medical history of mood and anxiety disorder, we selected nine matched in age and sex with the positive participants among persons without any medical history of mood and anxiety disorder as “controls” (Fig. 1): six men (67, 73, 77, 78, 79, and 84 years of age in the positive group; 70, 73, 75, 78, 79, and 85 years of age in the negative group) and three women (74, 77, and 87 years of age in the positive group; 73, 77, and 87 years of age in the negative group, Additional file 1). Additionally, the psychiatrist excluded those who were suspected of having psychiatric disorders other than MDD and anxiety disorder (e.g., dysthymic disorder, alcoholics, schizophrenia, and dementia) from both cases and controls.

### Collection of urine

Urine was collected and used to measure the urine-specific gravity (ATAGO pocket refractometer PAL-09S, Tokyo, Japan) and ten items (glucose, protein, bilirubin, urobilinogen, pH, blood, ketones, nitrite, leukocyte, creatinine, and color) using a multiparametric test strip (AUTION sticks 10PA, Arkray Inc., Kyoto, Japan), which was digitized using PocketChem (Arkray Inc.). Urine was frozen in liquid nitrogen for 30 min following collection. Urinary creatinine concentrations were measured using a LabAssay™ Creatinine Colorimetry Kit (Wako Pure Chemical Industries, Ltd. Osaka), based on the Jaffé method, as was serum creatinine (mg/dL) [38].

### Chemicals

Standard chemicals were used as follows: dimethyl sulfone (99.9% pure standard for quantitative NMR, catalog no. 41867, Sigma, MO, USA), phenethyl isothiocyanate (> 97% pure, catalog No. P0986, Tokyo Chemical Inc., Tokyo, Japan), 1-butene 4-isothiocyanate (> 96% pure, catalog No. I0443, Tokyo Chemical Inc.), dimethyl sulfoxide (99.5% pure, catalog No. 047-29353, Wako Pure Chemical Industries, Ltd.), hexanoic acid (> 98% pure, catalog No. H0105, Tokyo Chemical Inc.), 2,2,4-trimethyl-1,3-pentanediol monoisobutyrate (Texanol^Ⓡ^, catalog No. 40366, Alfa Aesar, Lancashire, UK), and *n*-alkane (C9-C40: 50 µg/mL; C10, 20, 30, and 40: 100 µg/mL, catalog No. 102158321, GLC Sciences Inc., Tokyo, Japan).

### Extraction of urinary VOCs by solid-phase microextraction

Urine was thawed on ice and centrifuged at 13 000 × *g* for 10 min. The supernatant was divided into 200 µL within a 2 mL glass vial packed tightly with a magnetic gold seal (PTFE/silicone septum 9 mm diameter × 1 mm thickness, GL Science, Tokyo, Japan), and disinfected at 65 °C for 15 min. Urinary VOCs in vials were extracted by headspace solid-phase microextraction (SPME) methods, in which the fiber was constructed with 2 cm 50/30 µM divinylbenzene/carboxen/polydimethylsiloxane (Supelco, Bellefonte, PA, USA). The SPME was preconditioned at 240 °C for 20 min and inserted into the headspace of the vial, including the urine. Finally, the VOCs in the headspace were extracted using SPME fiber at 45 °C for 60 min from the 18 urine samples (9 patients and 9 controls).

### GC–MS

The VOCs were detected by gas chromatography using a quadrupole mass spectrometer (QP-2010 Ultra, Shimadzu, Kyoto, Japan). The SPME fiber with the absorbed VOCs was inserted into the injection port of the GC and desorbed for 30 min at 240 °C. The injector port was maintained at 240 °C and a pulsed splitless injection was performed for 3 min. The GC–MS system was equipped with a 30-m HR-20 M column (column internal diameter 0.32 mm, film thickness 0.5 µm; Shinwa Chemical Industries, Ltd., Kyoto, Japan). We employed the following chromatographic protocol for separation before MS analysis: maintain at 40 °C for 10 min then heat at a ramp rate of 5 °C/min to 240 °C with a 10-min hold at the final temperature. Helium was flown at a constant linear velocity of 60 cm/s. The MS operating parameters were as follows: ion source temperature, 200 °C; interface temperature, 240 °C; ionizing energy, 70 eV; scanning frequency 5 Hz; and mass range 35–300 m*/z*. As described previously [28], we confirmed little difference in the absolute area (m/z 174) for *p*-bromofluorobenzene (standard solution 021-12041, Wako Pure Chemical Industries, Ltd.) between the SPME collection in C57Bl/6j urine (200 μL) including 100 ng of *p*-bromofluorobenzene and liquid injection (1 μL) of 100 ng of *p*-bromofluorobenzene by GC-MS. Furthermore, when we injected *p*-bromofluorobenzene collected by SPME at the beginning and end into multiple samples for GC–MS, we obtained the same absolute areas (m/z 174) between the beginning and end. Under the condition, we examined 18 samples sequentially, and the evaluation was performed twice on different days. The results obtained in two independent experiments showed the same trend.

### Data processing and statistics

Ion peaks generated from electron ionization were integrated into chromatographic peak areas (*m/z*) using the GC-MS Solution software (ver. 4.45; Shimadzu). Peak identification was accomplished by comparison with the retention time of standards using the NIST 14 standard reference database (NIST/EPA/NIH mass spectral library) and was confirmed with standard references of VOCs. Differential ion peaks between MDD and/or agoraphobia subjects and control subjects were extracted and identified using the following steps. The total ion current (TIC) was extracted based on the retention times of the ion peaks associated with *p* < 0.05, according to the GC-MS Solution software. In detail, TICs showing differential areas were extracted among 157 VOCs detected in this study (Additional file 2) as follows: (1) raw area of TIC from MDD/agoraphobia positive or negative urine was more than 10,000; (2) the TIC was detected in urine samples of at least three persons with or without MDD/agoraphobia, and (3) the average TIC area in MDD/agoraphobia positive urine samples showed > 1.5-fold increase or < 0.7-fold decrease as compared to the control urine samples. Metabolites in the TICs were identified by comparing the fragmentation patterns of ion peaks (*m/z*) and retention times with standards and *n-*alkane series. In addition, the XCMS software [39], package version 1.3.2 (http://masspec.scripps.edu) in R version 3.2.3 (http://cran.r-project.org/) was used to perform peak-matching, nonlinear retention time alignment, and quantitation of mass spectral ion intensities.

Texanol^Ⓡ^ was analyzed using GC-MS (QP-2010 Ultra, Shimadzu, Kyoto, Japan) with InertCap PureWAX (GL Science, Tokyo, Japan) [29] and using the ^1^H NMR spectra of compounds that were recorded on a JNM-ECS 400 spectrometer (JEOL Ltd., Tokyo, Japan) with tetramethylsilane as the internal standard (Additional file 3).

Descriptive statistics of the cohort data and ion peak (m/z) areas of VOCs are presented as the mean ± standard error of the mean; data with a non-normal distribution were analyzed using a non-parametric Mann–Whitney *U*-test, and *p*-values ≤ 0.05 were considered statistically significant. Moreover, we carried out Pearson’s or Spearman’s correlation analysis when adequate to investigate the association between combined VOCs and anxiety and depression symptoms.

We investigated whether differential amounts of VOCs could distinguish subjects with or without MDD and agoraphobia using a quadratic discriminant function in Excel and KyPlot 5.0 (KyensLab Inc., Tokyo, Japan).

Receiver operating characteristic (ROC) curve analyses were performed using GraphPad Prism 8 (GraphPad Software, San Diego, CA, USA) to determine the accuracy of each VOC in distinguishing subjects with and without MDD and agoraphobia symptoms. Furthermore, linear regression analyses performed in Excel and IBM SPSS Statistics 27.0.1 were used to determine, in each subject, the unstandardized predicted (PRE-1) values of two combined VOCs (texanol and texanol isomer), three combined VOCs (dimethyl sulfone, phenethyl isothiocyanate, and hexanoic acid), and five combined VOCs (dimethyl sulfone, phenethyl isothiocyanate, hexanoic acid, texanol, and texanol isomer). The ROC curves of the combined VOCs were then determined. Using an XY table, the correlations between the values of the combined VOCs and the cohort scores were assessed by Pearson’s *r* using Prism 8. The strength of the correlation was determined according to the guidelines put forth by Evans [40–42]. Furthermore, significant correlations were estimated using a curve fit with quadratic and cubic polynomial curves using Prism 8.

## Results

### Assessment of MDD and/or agoraphobia disorders and frailty

A psychiatrist diagnosed nine persons with MDD and/or *agoraphobia* disorders, and nine negative control persons among 639 elderly people included in The Otassha Study 2011 cohort 2015 survey (Fig. 1).

The assessments of MDD/agoraphobia and frailty are summarized in Table 1. Among the activity indices for the classification of frailty, the mean Kihon CL score was increased two-fold in positive persons with MDD and/or *agoraphobia* disorders compared to the negative persons. Positive persons also showed a 2.7-fold (*p* = 0.014, Mann–Whitney *U*-test) increase in the reported-by-others DSKC score and a 5.3-fold (*p* = 0.0002) increase in the self-reported DSKC score compared to the negative persons. There were no differences in the plasma and urinary creatinine values, urinary specific gravity, or urinary creatinine value per weight between negative and positive persons (Table 1).

**Table 1:** Comparisons of the cohort items between persons negative and positive for major depressive disorder and/or *agoraphobia* disorder

	Comparisons					Mann–Whitney *U*-test	
	Control (n = 9^a^)		MDD and/or agoraphobia (n = 9^a^)		Disorders/control		
Cohort items	Average	S.E.M	Average	S.E.M	Fold	*p* valued (two-tailed)	*p* valued (one-tailed)
Activity index for classification of frailty							
Kihon CL score^b^	3.667	0.943	7.222	1.164	1.97	0.0796	*0.0398
TMIG-IC^c^	12.00	0.527	11.22	0.572	0.94	0.3193	0.1596
JST-IC^d^	11.33	1.225	9.222	1.331	0.81	0.2829	0.1414
Depression-related test							
DSKC score^e^	1.000	0.441	2.667	0.289	2.67	*0.0142	**0.0071
DSKC self-score^f^	0.667	0.373	3.556	0.176	5.33	***0.0002	***0.0001
SDS score^g^	33.56	2.615	40.44	2.652	1.21	0.1405	0.0703
GRID-HAMD^h^	n.d	n.d	12.22	1.526	n.d	n.d	n.d
Biochemical assay							
Serum creatinine^i^	0.720	0.049	0.769	0.056	1.07	0.4240	0.2129
Urinary creatinine^i^	76.82	13.65	79.44	11.76	1.03	0.8123	0.4062
Urinary creatinine/weight^j^	1.344	0.199	1.564	0.300	1.16	0.8633	0.4317
Urinary specific gravity^k^	1.014	0.002	1.014	0.001	1.00	> 0.9999	0.5000

^a^Six men and three women comprised the nine positive persons with MDD and/or agoraphobia disorder, and their age and sex correspond with those of nine negative control persons

^b^The Kihon (checklist) CL score includes 20 items within the Kihon checklist as described by Kera et al. [14]

^c^Tokyo Metropolitan Institute of Gerontology Index of Competence (TMIG-IC) consists of a 13-item questionnaire that includes self-reliance, intelligence, and social participation [31]

^d^Japan Science and Technology Agency Index of Competence (JST-IC) consists of a 16-item questionnaire that includes use of new devices, intelligence, life management, and social participation [15]

^e^The depression section of the Kihon Checklist (DSKC) score consists of a five-item questionnaire within the Kihon checklist, assessed through report-by-others [32]

^f^Depression section of the Kihon Checklist (DSKC), using a self-report assessment method

^g^Self-rating Depression Scale (SDS) score [33]

^h^GRID-Hamilton Rating Scale for Depression (HAMD) for assessing the severity of the major depressive disorder (MDD) [13]; n.d. not determined

^i^Serum creatinine (mg/dL) and urine creatinine (mg/dL) were determined using the Jaffé method [38]

^j^Total creatinine weight (mg)/body weight (kg) per urination

^k^Urinary specific gravity was determined by refractive index measurement

### Determination of the urinary profiles of MDD and/or agoraphobia in older people using SPME GC–MS

**Fig. 2:**
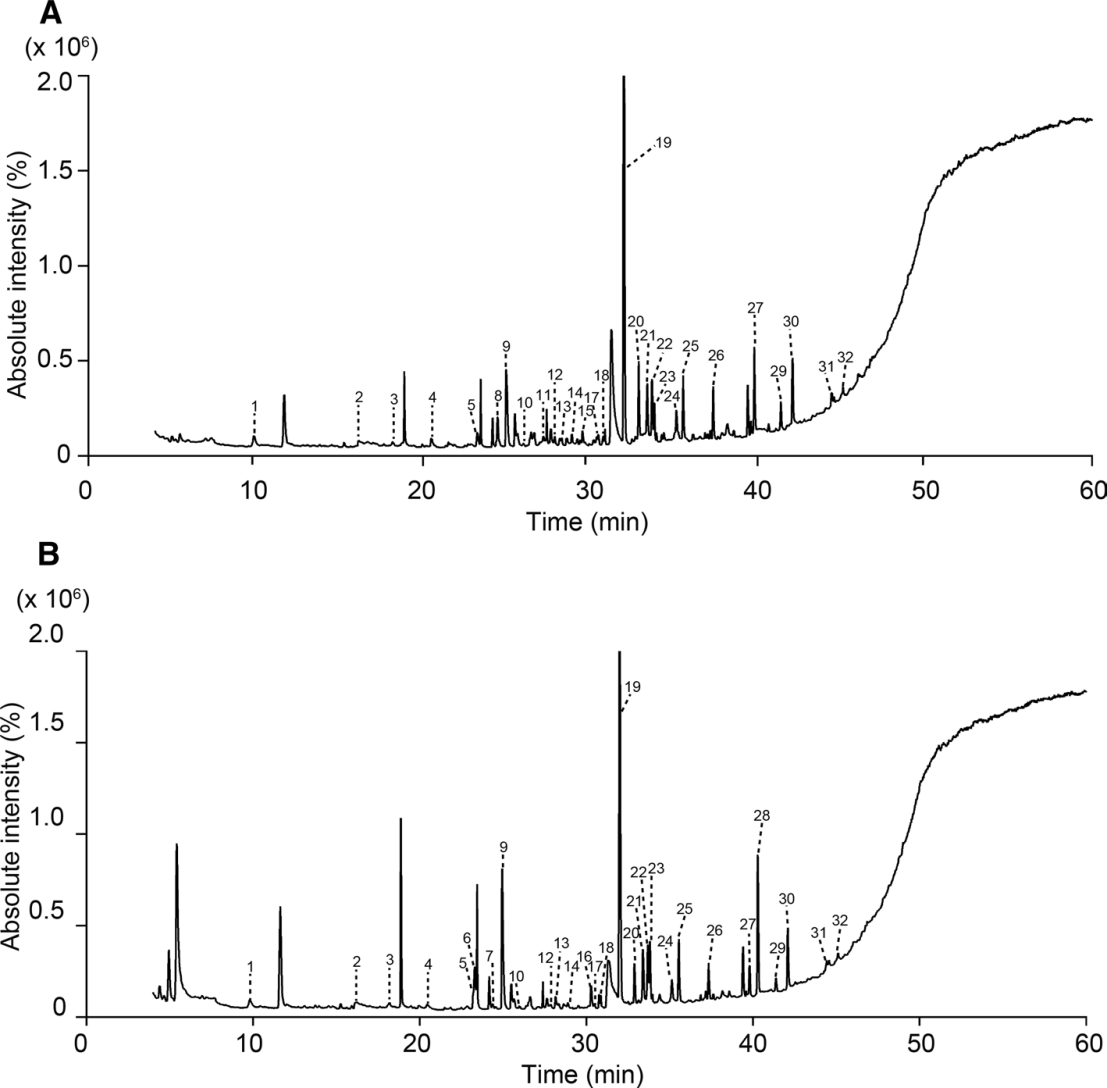
Typical gas chromatography–mass spectrometry chromatogram of total ion current of volatile organic compounds in the urine of a healthy control **a** and a patient with major depressive disorder **b** Total ion currents (TICs) were obtained from the analysis of the samples (200 µL) using headspace solid-phase microextraction (divinylbenzene/carboxen/polydimethylsiloxane, 50/30 µM, 2 cm) and gas chromatography-mass spectrometry **(**GC–MS) equipped with a ULBON HR-20 M capillary column (30 m, internal diameter 0.32 mm, thickness 0.5 µM). The extraction temperature was 45 °C, and the extraction time was 60 min. Desorption was performed at 240 °C for 3 min. The injection was pulsed splitless (closed, 3 min). The temperature program consisted of an initial temperature of 40 °C for 10 min, followed by an increase of 5 °C /min to 240 °C with the final temperature held for 10 min. The numbers indicate the metabolites whose similarity indices were > 85. 1: 4-heptanone; 2: 2-acetyl-2*H*-tetrazole; 3: 2-propanone, 1-hydroxy; 4: allyl isothiocyanate; 5: acetic acid; 6: 1-butene, 4-isothiocyanato-; 7: 1-hexanol, 2-ethyl-; 8: 1,3-benzodioxole, 5-propyl; 9: pyrrole; 10: DMSO; 11: 3-cyclohexen-1-ol, 4-methyl-1-(1-methylethyl)-; 12: butanoic acid; 13: cyclohexanol, 5-methyl-2-(1-methylethyl)-, [1S-(1.alpha.,2.alpha.,5.beta.)] compound; 14: butanoic acid, 3-methyl; 15: benzaldehyde, 2-chloro; 16: carvone; 17: pentanoic acid; 18: acetamide; 19: benzaldehyde, 2,4-dimethyl; 20: hexanoic acid; 21: 2,2,4-trimethyl-1,3-pentanediol 1-monoisobutyrate (texanol); 22: 2,2,4-trimethyl-1,3-pentanediol 3-monoisobutyrate (texanol isomer); 23: dimethyl sulfone; 24: hexanoic acid, 2-ethyl; 25: 1 dodecanol; 26: octanoic acid; 27: phenol, 2,5-dichloro; 28: phenethyl isothiocyanate; 29: decanoic acid; 30: phenol, 2,4-bis(1,1-dimethylethyl)-; 31: benzoic acid; 32: dodecanoic acid

Typical SPME GC–MS TIC chromatograms of urine samples of the positive and negative persons are shown in Fig. 2, indicating that very similar VOC profiles were obtained from the two groups of urine samples.

A total of 157 metabolites were detected using GC-MS (Shimadzu QP-2010 Ultra and TQ-8040; Additional file 2), including various chemical structures previously described in mice exhibiting epilepsy or anxiety- and depression-like behaviors [28, 29]. First, the peak areas of the TICs were manually compared between the two groups. In the 16 VOCs showing differential peak areas of the TIC, the fragment ion *m/z* values of the VOCs with the largest area within each fragmentation pattern were used to compare their absolute area values between the two groups of persons, resulting in the extraction of 2-acetyl-2*H*-tetrazole, dimethyl sulfone, and phenethyl isothiocyanate as potential biomarkers (*p* < 0.05, No. 1 to 16 in Table 2). Second, as the XCMS software [39] extracted four VOCs showing differential fragment ion *m/z* values between the two groups (No. 17 to 20 in Table 2), the fragment ion *m/z* values of the VOCs with the largest area were compared between the two groups of persons, resulting in the extraction of hexanoic acid, 2,2,4-trimethyl-1,3-pentanediol 1-monoisobutyrate (texanol) and 2,2,4-trimethyl-1,3-pentanediol 3-monoisobutyrate (texanol isomer) as additional biomarkers (*p* < 0.01).

**Table 2:** Absolute *m/z* values of volatile organic compounds and comparisons between negative and positive individuals for major depressive disorder and/or *agoraphobia* disorder

No	VOCs^a^	CAS No	Chemical class	Retention time (min)	Mainly observed *m/z*^b^	Used *m/z*^c^	Absolute area of *m/z*				Mann–Whitney *U*-test	
							Control (n = 9)		Depression & anxiety (n = 9)			
							Average	S.E.M	Average	S.E.M	*p* value^d^ (two-tailed)	*p* value^d^ (one-tailed)
1	2-acetyl-2*H*-tetrazole	51410-11-8	Diarylethers	15.97	42, 43	43	896,619	108,935	1,188,273	137,696	0.0939	*0.0470
2	2,3-Octanedione	585-25-1	Alpha-diketone	19.46	43, 57, 71	43	17,480	2462	31,273	8861	0.2973	0.1487
3	Allyl isothiocyanate	57-06-7	Organosulfur/isothiocyanate	20.50	41, 72, 99	99	667,199	554,308	171,145	54,542	> 0.9999	0.5000
4	Benzene, 1,3-bis(1,1-dimethylethyl)-	1014-60-4	Benzenoids	22.53	41, 57, 175	175	32,371	15,202	16,238	2147	0.3865	0.1933
5	Acetic acid	64-19-7	Carboxylic acids and derivatives	23.26	43, 45, 60	43	343,894	85,633	385,236	207,645	0.2224	0.1112
6	1-Butene 4-isothiocyanate^e^	3386-97-8	Organosulfur/isothiocyanate	23.39	55, 72, 113	113	29,243	14,845	1,306,697	1,191,445	0.1615	0.0807
7	Dimethyl sulfoxide^e^	67-68-5	Sulfoxides	25.99	45, 63, 78	63	32,197	6114	73,214	21,273	0.1135	0.0567
8	Menthol	15356-70-4	Prenol lipids	28.17	71, 81, 95	71	151,744	59,924	64,621	23,668	0.7304	0.3652
9	(-)-Carvone	6485-40-1	Prenol lipids	30.28	54, 82, 93	82	45,357	30,608	53,152	23,399	0.1903	0.0951
10	Dimethyl sulfone^e^	67-71-0	Sulfones	33.89	79, 94	79	660,288	140,962	1,328,412	290,284	0.0770	*0.0385
11	Benzothiazole	95-16-9	Benzenoids	35.05	82, 108, 135	135	19,864	3788	17,627	2854	0.7962	0.3981
12	Phenol	108-95-2	Benzenoids	36.12	65, 66, 94	94	43,106	19,370	39,801	14,563	0.6665	0.3332
13	*p*-Cresol	106-44-5	Benzenoids	37.62	77, 107, 108	107	31,128	11,066	65,472	36,074	0.4363	0.2181
14	2-Methoxy-4-vinylphenol	7786-61-0	Benzenoids	39.79	107, 135, 150	150	81,457	29,571	35,448	10,739	0.6665	0.3332
15	2,5-Dichlorophenol^e^	583-78-8	Benzenoids	39.86	63, 162, 164	162	168,307	57,814	1,041,674	587,894	0.1359	0.0680
16	Phenethyl isothiocyanate^e^	2257-09-2	Benzenoids/isothiocyanate	40.30	91, 163	91	31,162	8863	350,507	179,957	**0.0078	**0.0039
17	Hexanoic acid^e^	142-62-1	Medium-chain fatty acids	32.89	60, 73, 87	60	429,620	32,655	301,160	30,979	*0.0106	**0.0053
18	2,2,4-trimethyl-1,3-pentanediol 1-monoisobutyrate (Texanol)^e,f^	25265-77-4	Hydrocarbon	33.41	56, 71, 89	71	293,552	7765	355,679	9990	**** < 0.0001	**** < 0.0001
19	2,2,4-trimethyl-1,3-pentanediol 3-monoisobutyrate (Texanol isomer)^e,f^		Hydrocarbon	33.70	43, 71, 83	71	345,878	10,438	409,732	12,109	**0.004	**0.002
20	2-Piperidinone	675-20-7	Piperidine	38.21	55, 70, 99	99	107,248	47,157	93,972	43,154	0.9314	0.4657
21	Creatinine (mg/dL)^g^						76.82	13.65	79.44	11.76	0.8123	0.4062

Volatile organic compounds (VOCs) 1–16 were manually nominated from peaks of total ion current (TIC); VOCs 17–20 were nominated based on XCMS analyses showing differential quantities between the urine of positive (n = 9) and negative (n = 9) persons

^a^VOCs were determined by retention indices, which are relative retention times normalized to closely eluting *n*-alkanes on HR20M

^b^Three main peaks of *m/z* were exhibited here

^c^The area of a clearly detected *m/z* peak was used for the quantification of VOCs in urine

^d^Mann–Whitney *U*-test

^e^VOCs were confirmed using commercial standard references

^f^Two species of VOCs were determined using the ^1^H NMR spectra of the compounds (Additional file 3)

^g^Concentration of creatinine was determined as per Bonsnes and Taussky [38]

### Discrimination of persons with or without MDD and/or agoraphobia symptoms using urinary VOCs

We investigated whether the urinary VOCs described in Table 2 could be used as biomarkers in a screening test for MDD and/or agoraphobia symptoms.

**Fig. 3:**
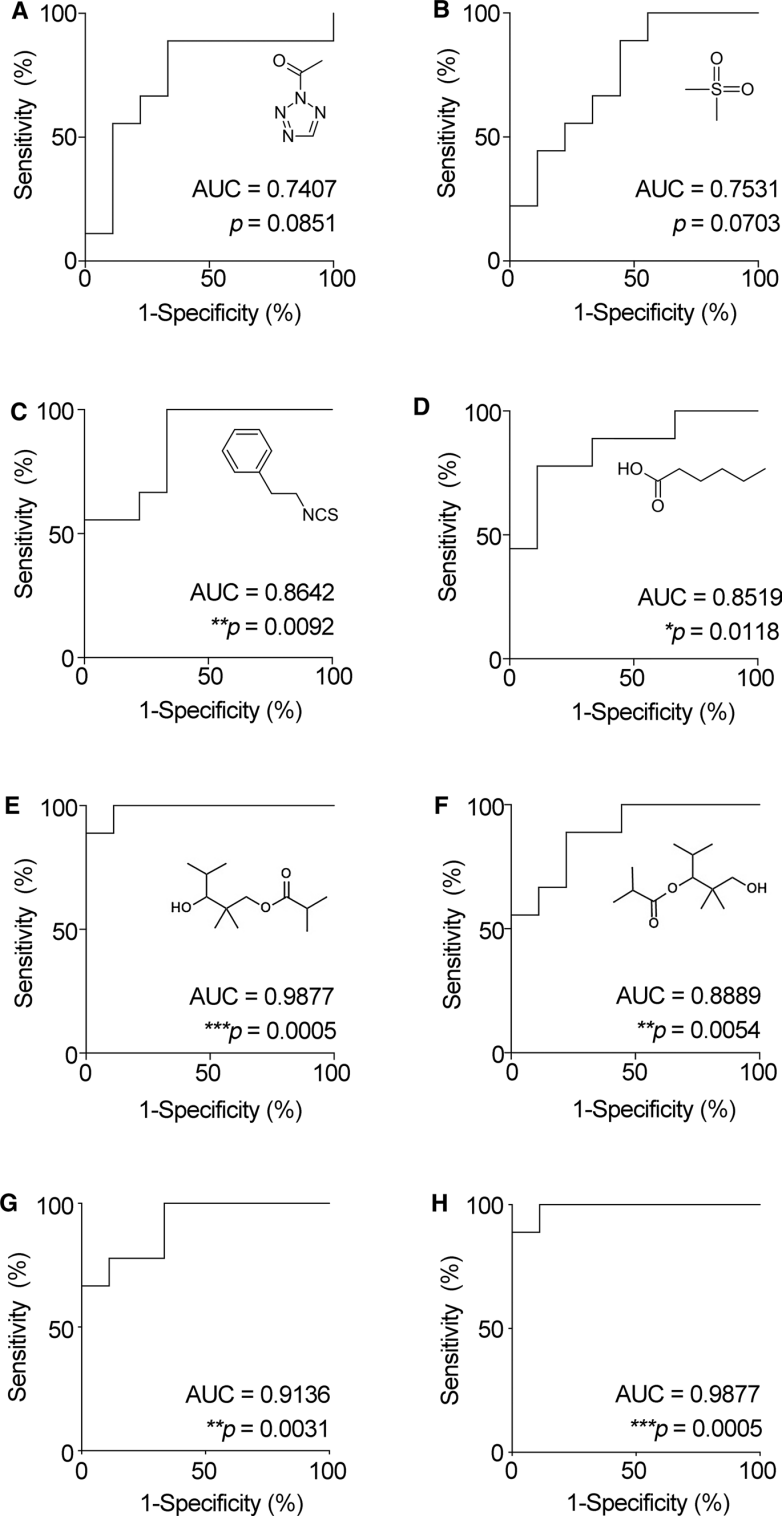
Receiver operating characteristic curves showing the differential amounts of urine volatile organic compounds between the two diagnostic groups. **a** 2-acetyl-2*H*-tetrazole; **b** dimethyl sulfone; **c** phenethyl isothiocyanate; **d** hexanoic acid; **e** 2,2,4-trimethyl-1,3-pentanediol 1-monoisobutyrate (texanol); f 2,2,4-trimethyl-1,3-pentanediol 3-monoisobutyrate (texanol isomer); **g** combined index of three volatile organic compounds (VOCs) (dimethyl sulfone, phenethyl isothiocyanate, and hexanoic acid); **h** combined index of two VOCs (texanol and texanol isomer). *AUC* area under the curve

A quadratic discriminant function indicated that six VOCs were able to fully distinguish MDD and/or agoraphobia-positive persons from negative persons, with significantly different covariance in Box’s M test (*p* = 0.001) and a discriminant probability of 100% (Table 3). To investigate which VOCs and VOC combinations could represent useful biomarkers, we performed an ROC curve analysis. Fitting ability was visualized by the ROC curve and evaluated by the area under the curve (AUC), resulting in the detection of highly predicting VOCs and VOC combinations (AUC > 0.7; Additional file 4). In particular, phenethyl isothiocyanate, hexanoic acid, texanol, and texanol isomer exhibited significantly higher predictive power, with AUC values of 0.8642, 0.8519, 0.9877, and 0.8889, respectively (Fig. 3). No false negatives were predicted using phenethyl isothiocyanate and texanol (sensitivity = 1; Additional file 4), indicating that these VOCs are useful biomarkers for the initial screening of MDD and agoraphobia symptoms. We also performed an ROC curve analysis of combined VOC indices using unstandardized predicted (PRE-1) values of combined VOCs in each participant for linear regression analyses. 2-acetyl-2*H*-tetrazole was excluded because it was not confirmed with the standard chemical by GC–MS. Finally, the combined indices of three VOCs (dimethyl sulfone, phenethyl isothiocyanate, and hexanoic acid) and two VOCs (texanol and texanol isomer) exhibited remarkably high predictive power, with AUC values of 0.9136 and 0.9877, respectively (Fig. 3, Additional file 4). The correlations between the values of texanol and texanol isomer were assessed using Pearson’s *r*: *r* = 0.8094 (*p* = 0.0082) in MDD and/or agoraphobia-positive persons and *r* = 0.5481 (*p* = 0.1266) in negative persons (Additional file 5). This indicates that MDD and/or agoraphobia disorders caused both texanol and texanol isomer to increase cooperatively; however, there was no correlation in the healthy elderly. Hence, the predictive power of two VOCs was more stable than that of single VOCs.

**Table 3:** Discriminant function analysis for determining membership of volatile organic compounds associated with major depressive disorder and/or agoraphobia

A. Coefficient for formula^#$^ with six volatile organic compounds								
Linear and constant terms		Quadratic terms	b1	b2	b3	b4	b5	b6
a1: 2-acetyl-2*H*-tetrazole	4.832E−05	b1	− 1.041E−11	6.300E−12	5.600E−11	− 4.000E−11	1.905E−10	− 2.000E−10
a2: Dimethyl sulfone	− 3.924E−05	b2	6.336E−12	1.500E−11	1.500E−10	4.700E−11	− 3.546E−10	2.530E−10
a3: Phenethyl isothiocyanate	− 6.685E−04	b3	5.626E−11	1.500E−10	2.700E−09	5.900E−10	− 1.718E−09	1.020E−09
a4: Hexanoic acid	1.356E−04	b4	− 4.193E−11	4.700E−11	5.900E−10	− 7.000E−11	5.154E−10	− 6.600E−10
a5: Texanol	− 3.441E−04	b5	1.905E−10	− 4.000E−10	-2.000E−09	5.200E−10	− 1.099E−09	1.640E−09
a6: Texanol isomer	3.574E−04	b6	− 1.956E−10	2.500E−10	1.000E−09	− 7.000E−10	1.641E−09	− 1.400E−09
a0	− 4.838E + 01							

B. Scores, discriminant probabilities, and errors calculated with six volatile organic compounds			
"Positive"evaluated by cohort items		"Negative" evaluated by cohort items	
Person no.	Score	Person no.	Score
1	3.031	10	− 12.478
2	76.077	11	− 38.054
3	13.281	12	− 19.532
4	3562.766	13	− 4.053
5	203.738	14	− 7.1304
6	16.322	15	− 18.572
7	21.570	16	− 13.173
8	14.522	17	− 8.5484
9	4521.694	18	− 13.075
% Discriminant probability	100.000	% Discriminant probability	100.000
% Error	0.000	% Error	0.000

Discriminant scores were calculated using the formula in A

*2H* 2-acetyl-2*H*-tetrazole, *DS* dimethyl sulfone, *2PI* phenethyl isothiocyanate, *HA*, hexanoic acid, *T* texano, *TI* texanol isomer, *VOC* volatile organic compound

#Score = a0 + (2H) * {a1 + (b1*b1*(H2) + b1*b2*(DS) + b1*b3*(2PI) + b1*b4*(HA) + b1*b5*(T) + b1*b6*(TI))} + (DS)*{a2 + (b2*b1*(H2) + b2*b2*(DS) + b2*b3*(2PI) + b2*b4*(HA) + b2*b5*(T) + b2*b6*(TI))} + (2PI)*{a3 + (b3*b1*(H2) + b3*b2*(DS) + b3*b3*(2PI) + b3*b4*(HA) + b3*b5*(T) + b3*b6*(TI))} + (HA)*{a4 + (b4*b1*(H2) + b4*b2*(DS) + b4*b3*(2PI) + b4*b4*(HA) + b4*b5*(T) + b4*b6*(TI))} + (T)*{a5 + (b5*b1*(H2) + b5*b2*(DS) + b5*b3*(2PI) + b5*b4*(HA) + b5*b5*(T) + b5*b6*(TI))} + (TI)*{a6 + (b6*b1*(H2) + b6*b2*(DS) + b6*b3*(2PI) + b6*b4*(HA) + b6*b5*(T) + b6*b6*(TI))}

^$^Judgement ("Positive" (1) or "Negative" (2)) = IF(COUNT((2H), (DS), (2PI), (HA), (T), (TI))—6,”?”, IF(Score >  = 0,1,2)), in which a person suffering from “MDD and/or agoraphobia,” as evaluated by the cohort items (Table 1), was judged as “positive” without error (0%), as described in B

**Fig. 4:**
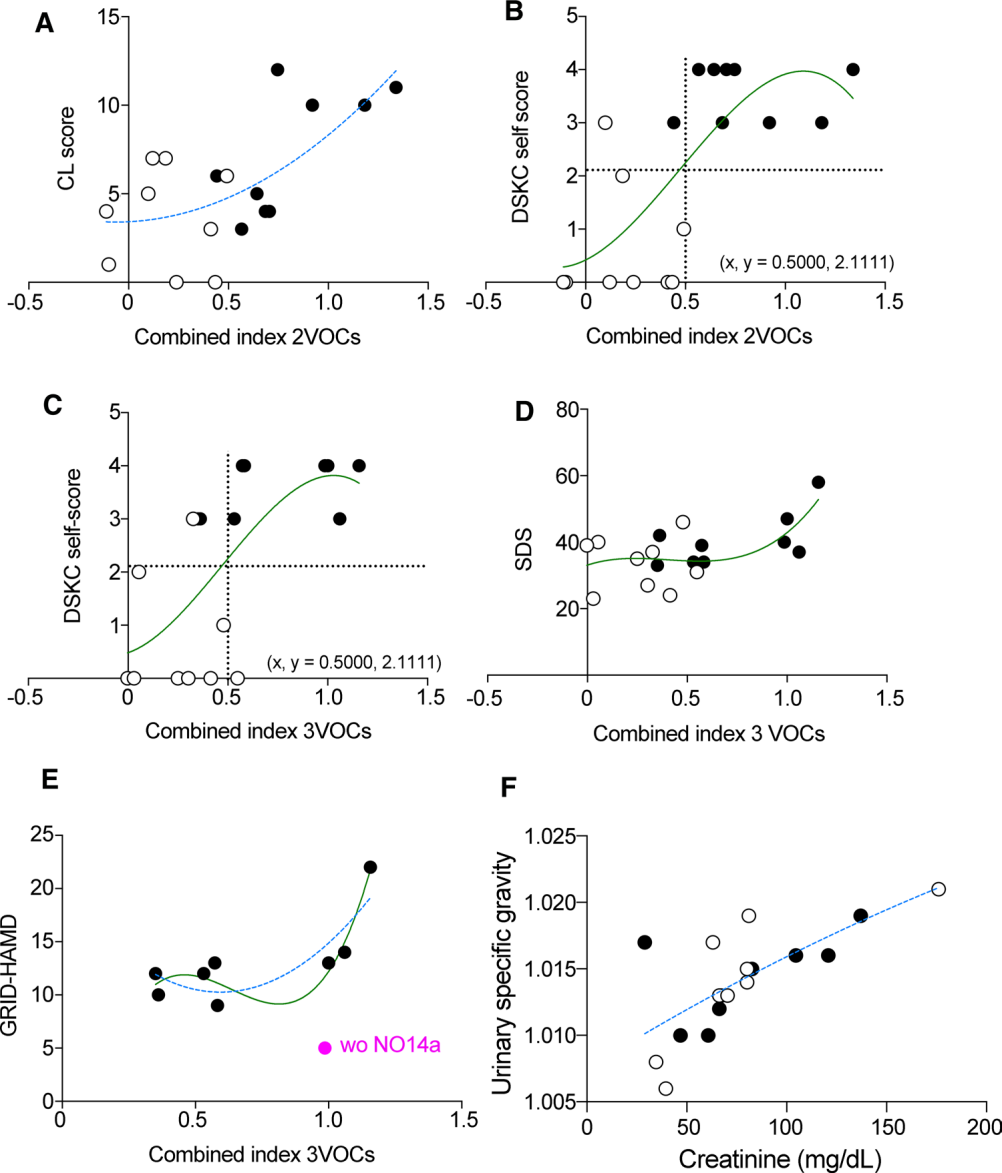
Curve fit to estimate the relationship between cohort assessments and combined three- or two-volatile organic compound indices. The curve estimations showed significant Pearson's correlations between cohort assessments and combined volatile organic compound (VOC) indices. Major depressive disorder and/or agoraphobia positive (black circles) and negative persons (white circles). Quadratic (dotted blue lines) and cubic (green lines) polynomial curves were fitted. The formulae and the corresponding *r*-values and *p*-values were analyzed using Prims 8 and SPSS, respectively. Formulae, *r- and p*-values: Y = 3.423 + 0.560*X + 4.335*X^2^, *r* = 0.660, F(2, 17) = 5.781, *p* = 0.014 in (**a**); Y = 0.420 + 1.853*X + 5.567*X^2^-3.924*X^3^, *r* = 0.709, F(3, 17) = 4.708, *p* = 0.018 in (**b**); Y = 0.483 + 1.317*X + 6.894*X^2^-4.882*X^3^, *r* = 0.661, F(3, 17) = 3.621, *p* = 0.040 in (**c**); Y = 33.109 + 19.639*X-58.487*X^2^ + 48.613*X^3^, *r* = 0.637, F(3, 17) = 3.190, *p* = 0.057 in (**d**); Y = 20.150–33.282*X + 28.014*X^2^, *r* = 0.839, F(2, 7) = 5.964, *p* = 0.047 in (**e**) (blue dotted line); Y = -13.614 + 137.017*X-233.638*X^2^ + 122.438*X^3^, *r* = 0.945, F(3, 7) = 11.033*, p* = 0.021 in (**e**) (green line); Y = 1.008 + 9.312e−005*X-9.005e−008*X^2^, *r* = 0.709, F(1, 17) = 16.138, *p* = 0.001 in (**f**). The black dotted lines show the mean values in the vertical and horizontal lines in (**b**, **c**). While the correlations in (**a**–**d**) and (**f**) included a subject (No. 14a) exhibiting agoraphobia but no depressive symptoms, the correlation in (**e**) did not include this subject. *Kihon CL score* Kihon Checklist that consists of 20 items [14]; *SDS* Zung Self-Rating Depression Scale, *GRID-HAMD* GRID-Hamilton Rating Scale for Depression

Pearson’s bivariate correlation analysis revealed relationships between the combined VOC indices and several cohort data for persons with or without MDD and/or agoraphobia symptoms (Additional file 6). The combined index of two VOCs correlated with the Kihon CL score [14] and DSKC self-score [32], while the combined index of three VOCs correlated with the DSKC self-score, SDS [33], and GRID-HAMD [13]. Curve fit estimation further indicated correlations between the combined VOCs and the cohort scores. The Kihon CL score was strongly correlated with the combined index of two VOCs (Fig. 4 a); the DSKC self-score was strongly correlated with the combined indices of both two and three VOCs (Fig. 4b, c); the SDS and GRID-HAMD scores were respectively strongly and very strongly correlated with the combined index of three VOCs (Fig. 4d, e). Among the well-known correlations, that between urinary creatinine and urinary specific gravity was also strong (Fig. 4f).

## Discussion

This study aimed to identify biomarkers of MDD and/or anxiety and frailty in elderly individuals using various combinations of VOCs. Our results provide the possibility that texanol and a texanol isomer are biomarkers for the screening and assessment of frailty, and that the combination of the dimethyl sulfone, phenethyl isothiocyanate, and hexanoic acid VOCs could be used to accurately estimate the MDD and agoraphobia levels of older people. As present results were derived from a small sample and this was an initial screening, performance using a larger sample size is needed for generalizing the utility of these identified biomarkers.

In this study, the Kihon CL [14], TMIG-IC [31], and JST-IC [15] were used to assess frailty. The CL score was only high in persons with MDD and/or agoraphobia symptoms. The TMIG-IC and JST-IC scores are indices of higher-level functional capacity, which tend to evaluate sociality-related life function [15, 31]. The TMIG-IC is composed of instrumental self-maintenance, intellectual activity, and social role domains and can assess deficiencies of instrumental activities of daily living (IADL) for which support and care are needed [43]. The JST-IC consists of four subscales—social engagement, technology usage, information practice, and life management—which require even higher functional capacity than those assessed by the TMIG-IC. There was little difference in the TMIG-IC and JST-IC scores between persons with and without MDD and/or agoraphobia, suggesting that the participants maintained their higher-level functional capacity without IADL disability [43]. Conversely, the CL score indicates the risk of frailty, which precedes IADL disability [43]. The CL score is reported to be valid in comparison with Fried’s physical frailty indices; the cut-off point for Fried’s frailty index is five points per maximum score of 20 [14]. Hence, this result suggests that the present participants with MDD and/or agoraphobia symptoms (average = 7.2) were defined as frail.

The Kihon CL used in this study [14], which was composed of 20 items, excluded the depression subdomain of the 25-item Kihon checklist [34]. The DSKC was independently utilized to determine the degree of depression using self-report assessment methods, as Fujisawa et al. [32] have reported its validity for this purpose. Indeed, both DSKC scores were high among the positive persons in the present study. However, Table 1 shows that the sensitivity achieved by report-by-others was lower than that of the self-report score, because the results of reporting-by-others questionnaires can vary depending on the circumstances and the interviewers [2]. However, there was little difference in the SDS between participants with and without MDD and/or agoraphobia symptoms. It has been suggested that older people tend to score higher than younger people, as the questionnaire used in the SDS contains somatic complaints [44]. This study confirmed that the control group did not show depressive and agoraphobia symptoms; hence, healthy elderly people tended to have high scores.

Physio-anatomical studies [45] have shown that depressive behavior is correlated with hypermetabolism of the subgenus cingulate cortex and amygdala, and hypometabolism of the dorsal prefrontal cortex and striatal regions [46], while the central nucleus of the amygdala (Ce) and bed nucleus of the stria terminalis (BST) are involved in organizing defensive responses to fear and anxiety [47]. These brain regions belong to the limbic system, including the hypothalamus, which is involved in autonomic and hormonal regulation. Furthermore, pharmacological and pathological analyses suggest monoamine associations in MDD and anxiety disorders. The monoamines– serotonin, dopamine, and noradrenaline are produced in the midbrain dorsal raphe nucleus, ventral tegmental area, and locus coeruleus, respectively. These brain regions are known to respond to functional amygdala hypersensitivity [46, 48]. However, little evidence implicates monoamine neurotransmission in the development of MDD and anxiety. Therefore, drugs related to monoamines seem to restore mood by modulating processes unrelated to the primary causative factors of MDD and anxiety. Numerous studies suggest that depression and anxiety are associated with alterations in various biological functions [46]. Recent reports suggest that non-coding RNA [49, 50], microbiota [51], diet-derived signaling molecules [52], and maternal immune activation [53] affect the development of depression and anxiety. Among them, studies have especially focused on the intestinal microbiota secreting GABA, tryptophan, and short-chain fatty acids of small metabolites [54], with the developmental crosstalk between intestinal microbiota and hypothalamus–pituitary–adrenal (HPA) axis [55], and inflammatory-response molecules [56].

It is known that the components of odorous VOCs excreted from the human body reflect the metabolic conditions of the individual. Hundreds of VOCs are initially produced in various cells inside the body and secreted as final metabolic products into urine, feces, breath, sweat, and vaginal secretions [26]. Blood also contains VOCs, which are eventually emitted into the breath, sweat, etc. Specific VOCs have been suggested to be useful in the olfactory diagnosis of several disorders [57] including infectious diseases [58], inherited metabolic disorders [26], and cancer [59, 60]. To extract the VOCs specific to a given disease, it is best to collect emitted samples non-invasively and efficiently. In neuropsychiatric disorders, multiple VOCs in the breath of patients with Alzheimer's, Parkinson's disease, and schizophrenia have been assessed for their utility as biomarkers [61, 62]. While VOCs in the breath of patients with MDD and anxiety as biomarkers have been less reported, in 2020 [63], machine learning analysis of an electronic nose study showed no such association stated above. Recently, breath VOCs in MDD patients and healthy controls were investigated by a novel approach using proton-transfer-reaction mass spectrometry (PTR-MS) in a small-sized sample, and several mass concentrations (m/z) were identified as biomarkers of MDD. As all m/z are not related to known compounds [64], the knowledge of these compounds is awaited and is expected to open new avenues for understanding the metabolic changes they may be involved in.

In this study, it was difficult to collect the breath and sweat of most animals to compare human and experimental animal models in terms of metabolism-related VOCs. Additionally, VOCs are required to volatilize under specific conditions, without spontaneous volatilization. Here, urine was selected to analyze VOCs as potential biomarkers of MDD and/or agoraphobia in older people, and the VOCs volatilized from urine treated at 45 °C for 1 h were extracted by the SPME fiber and detected by GC–MS. Using this method, we previously profiled urinary VOCs in a mouse model of TLE induced by amygdala stimulation [28], and in a mouse model of anxiety-depression produced by a deficiency of sialyltransferase St3gal4 [29]. The TLE mouse model was associated with 13 VOCs, among which methanethiol, disulfide, dimethyl, and 2-butanone were strongly predictive biomarkers [28]; while older (age 20 − 35 weeks) St3gal4-deficient mice with anxiety-depression were associated with 12 VOCs, among which trimethylamine, 3-penten-2-one, benzaldehyde, beta-farnesene, and texanol isomer were predictive biomarkers [29]. Here, we used the same method in humans, screening urinary VOCs in the elderly with MDD and/or anxiety disorder. We nominated 20 types of VOC, including VOCs not showing significance because of large variance among positive and negative control participants. The 20 types included six VOCs, namely 2-acetyl-2*H*-tetrazole, dimethyl sulfone, phenethyl isothiocyanate, hexanoic acid, texanol, and texanol isomer, that exhibited statistical significance as biomarkers of MDD and/or agoraphobia in the elderly. Using ROC curve analysis and Pearson’s bivariate correlations, we found that two combinations of multiple VOCs were effective as biomarkers. A group of three VOCs (dimethyl sulfone, phenethyl isothiocyanate, and hexanoic acid) was strongly associated with the GRID-HAMD score, thus representing a biomarker of MDD in the elderly. Another group, composed of texanol and texanol isomer, was strongly related to the Kihon CL score, and thus a biomarker of frailty.

Next, we investigated the metabolic pathways with which these five VOCs are associated and assessed how they may be associated with MDD and/or agoraphobia in the elderly and other symptoms in humans and experimental mice. First, the levels of volatile sulfur compounds, dimethyl sulfone, and phenethyl isothiocyanate were increased in the urine of older people with MDD and/or agoraphobia. Dimethyl sulfone is persistently present in the human metabolome under certain conditions. In a 2014 review, He and Slupsky [65] summarized a metabolic pathway that is initiated with the microbial metabolism of methionine that produces methanethiol, followed by methylation to dimethyl sulfide in the host colon, and by unknown conversions of dimethyl sulfide to dimethyl sulfoxide. Dimethyl sulfoxide is then efficiently absorbed into the bloodstream and metabolized into dimethyl sulfone without catabolism by the intestinal microbiota. It was observed that dimethyl sulfoxide tended to increase in subjects with MDD and/or agoraphobia, likely as observed in the increase of dimethyl sulfone, because dimethyl sulfone is more stable than dimethyl sulfoxide as a metabolic end-product in urine [65]. A mouse model of epilepsy produced by amygdala-kindling stimulation showed decreases in the levels of methanethiol in the urine [28]. These results suggest that the methionine-methanethiol-dimethyl sulfone metabolic pathway functions via combinations of host and microbiota reactions related to the limbic system, including the amygdala, in humans and mice.

Phenethyl isothiocyanate is a potent cancer chemopreventive drug acting as an inactivator of some cytochrome P450s, including CYP2E1, which is involved in the bioactivation of carcinogens. For example, a reactive sulfur atom generated during the desulfurization of phenethyl isothiocyanate participates in the inactivation of CYP2E1 [66]. As the inactivation of CYP2E1 induces an increase in dopamine in rat substantia nigra [67], an increase in phenethyl isothiocyanate could affect brain dopamine levels via decreased CYP2E1 levels. Furthermore, phenethyl isothiocyanate is known to covalently modify the cysteine side chains of glutathione S-transferase, which irreversibly inhibits enzymatic activity [68]. The 20 VOC types shown in Table 2 included 3 VOCs (allyl isothiocyanate, 1-butene 4-isothiocyanate, and phenethyl isothiocyanate) that are isothiocyanates produced naturally from cruciferous plants. It is known that these three isothiocyanates are adsorbed into mammalian cells [69], conjugated with glutathione by glutathione-S-transferase, and transformed into cysteinyl glycine by gamma-glutamyl transpeptidase. The latter is *N*-acetylated to form mercapturic acid, which is excreted into the urine [70–72]. Furthermore, it has been reported that glucosinolate derived from cruciferous plants is converted into isothiocyanate by the myrosinase enzyme in the microbiota of the gastrointestinal tract [73] and that 1-butene 4-isothicyanate is a hydrolytic product of glucosinolate [74]. The present study indicated that 1-butene 4-isothiocyanate (weakly) and phenethyl isothiocyanate (strongly) were detected without degradation or conjugation as urinary VOCs in the elderly with MDD and agoraphobia. This suggests that older people with MDD and anxiety experience poorer metabolism of isothiocyanates, particularly phenethyl isothiocyanate, including aromatic rings.

Benzyl isothiocyanate, including an aromatic ring, was not detected as a differential VOC (Table 2). However, Fujita et al. [29] demonstrated that benzaldehyde is one of the VOCs that was increased among urinary VOCs in St3gal4-deficient mice with anxiety and depression compared to littermate wild-type mice. Benzaldehyde is a metabolite of benzyl isothiocyanate formed by cytochrome P450 [75]. Hence, it is proposed that a similar metabolic pathway via isothiocyanate is involved in depression and anxiety in humans and mice.

Hexanoic acid is a short-chain fatty acid (SCFA), which is the main metabolite produced in the large intestine through the anaerobic fermentation of indigestible polysaccharides [76]. SCFAs can cross the blood–brain barrier and are found in human CSF [77]. It has also been suggested that SCFAs positively affect emotion, cognition, and the pathophysiology of brain disorders, including the inhibition of depression-like behavior [78]. This study indicated that hexanoic acid levels were reduced in the urine of older people with MDD and/or agoraphobia, suggesting that in these persons the amount of hexanoic acid produced by the microbiota may be reduced, or that they may require more hexanoic acid to recover from depression. The mouse model of epilepsy produced by amygdala-kindling stimulation showed increased 2-pentanone in the urine [28]. Given the possibility that 2-pentanone is produced from hexanoic acid by the microbiota [79], increased 2-pentanone may be induced by an increase in hexanoic acid in these mice. This suggests that the hexanoic acid-2-pentanone metabolic pathway of the microbiota is related to the limbic system, including the amygdala, in both humans and mice.

The amounts of texanol and the texanol isomer were associated with the Kihon CL scores evaluating general life functions in older people. Texanol^Ⓡ^ is the most commonly used coalescing agent in latex paint and is a mixture of 2,2,4-trimethyl-1,3-pentanediol 1-monoisobutyrate (texanol) and 2,2,4-trimethyl-1,3-pentanediol 3-monoisobutyrate (texanol isomer) [80]. 2,2,4-Trimethyl-1,3-pentanediol monoisobutyrate is one of the VOCs used for tracing indoor and urban air pollutant levels [81, 82], and it is known that texanol isomer is semi-volatile and continues to be emitted from latex-painted surfaces for months after application [80]. We confirmed the 60:40 ratio in the Texanol^Ⓡ^ solvent (Additional file 3), while the mean ratios of urinary texanol and texanol isomer were 45.9:54.1 and 46.5:53.5 in control and depression subjects, respectively. This suggests that the texanol isomer, being semi-volatile, is more persistent in urine than texanol. In a 1997 review, Nielsen et al. [83] summarized a metabolic pathway in which the hydrolysis of texanol forms isobutyrate and trimethyl pentanediol, then free 2,2,4-trimethylpentane-1,3-diol (TMPD) and 2,2,4-trimethyl-1,3-hydroxyvaleric acid as well as their glucuronides and sulfates, and eventually isobutyrate is detected in the urine. Here, we detected the presence of texanol in the urine without its hydrolysis. Weschler et al. [84] proposed the possibility that the dermal pathway allows texanol to be transported into human blood or urine directly. This suggests that older people with reduced liver metabolism who ingest texanol from the environment via inhalation and the skin have reduced life function, as indicated by the Kihon CL score, and tend to exhibit MDD and/or agoraphobia symptoms. Fujita et al. [29] demonstrated that the texanol isomer is a predictor of depression and anxiety in older St3gal4-deficient mice (aged 20 − 35 weeks) with anxiety-depression. This suggests that the xenobiotic metabolic pathway is similar between older humans and mice showing depressive and anxiety symptoms.

The medication lists recorded in the participants’ medication handbooks (Additional file 1) did not contain any drugs taken only by the persons with MDD and/or agoraphobia, and the six urinary VOCs were not derived from the degradation of the medications shown in the list. Overall, the urinary differential VOCs we identified suggest that depression and anxiety and frailty levels could be associated with metabolic changes, both endogenous and in the gut flora. In particular, it was proposed that four VOCs, namely dimethyl sulfone, phenethyl isothiocyanate, and texanol/texanol isomer, were respectively derived from microbiota, plants, and the environment, and were detected directly in urine following xenobiotic metabolism. Hence, it is suggested that persons with MDD and/or agoraphobia exhibit a specific decrease in the xenobiotic metabolic activity of the four VOCs. Additionally, we analyzed whether VOCs were influenced by diet, alcohol, tobacco, and sleep condition (Additional file 7). Pearson’s bivariate correlation analysis revealed that in individuals with MDD and/or agoraphobia, two combined VOCs did not correlate with sleep condition and eating frequencies of any diet, as shown in Additional file 8; however, a positive correlation to alcohol consumption and frequency of the drinking per week was observed (Additional file 9(c), (d)). Thus, alcohol consumption may worsen the metabolism of texanol and texanol isomer and may accelerate the progress of frailty. The combination of three VOCs (dimethyl sulfone, phenethyl isothiocyanate, and hexanoic acid) correlated negatively with frequencies of green and yellow vegetables and seaweed consumption in individuals with MDD/agoraphobia (Additional file 9(g), (h)). People suffering from MDD/agoraphobia with a preference for plant foods tended to have higher concentrations of the three VOCs and severe symptoms of the disorders. In all people with and without MDD and/or agoraphobia, the frequency of eating fats and oils was correlated with both 2 and 3 VOC combinations, suggesting the possibility that the higher the intake, the higher the metabolism of VOCs. Additionally, the metabolism of the 2 VOC combination may be enhanced in healthy elderly individuals by eating soy products (Additional file 9(a)). Finally, among the healthy elderly, 3 VOCs tended to be lower in males as compared to the females (Additional file 9(e)); however, there were no differences between 2 or 3 VOCs among females and males with MDD/agoraphobia disorders. Furthermore, there was no correlation between 2 or 3 VOCs and age (Additional file 8).

In the Otassha Study 2011 cohort’s 2015 survey, a DSKC score of ≥ 2 was used to screen for mood disorders before identification of 8 MDD by a psychiatrist. The psychiatrist also determined whether persons with a DSKC score ≥ 2 suffered from an anxiety disorder and identified 3 persons with agoraphobia. As the survey did not perform the initial screening for anxiety, the persons with agoraphobia identified by the psychiatrist may not be representative of participants in the 2015 survey. Under the condition, present study aimed to identify novel urinary VOC biomarkers of MDD and agoraphobia. However, urinary end-product VOCs suggest that MDD and agoraphobia may share a common metabolic pathway.

The study has some limitations owing to a small sample size comprising community-dwelling elderly, and therefore, the biomarkers of MDD/agoraphobia cannot be generalized to the elderly in other geographical areas or those in different age groups. Other major limitations arise due to semi-quantitative analysis and a lack of prospective evaluation of MDD and/or anxiety disorder. Hence, we plan on a prospective evaluation of MDD and/or anxiety disorder including agoraphobia biomarkers using urine samples collected through follow-up studies since 2015 in the near future. Additionally, elucidating the involvement of peripheral molecules in specific metabolic cascades related to VOC biomarkers in the central nervous system is necessary.

In conclusion, the present results suggest that urinary VOCs, as detected by SPME GC-MS, can be metabolic biomarkers of MDD and/or agoraphobia and the frailty status of elderly people. The results also suggest that urinary VOCs in the elderly with MDD and/or agoraphobia belong to metabolic pathways similar to those of urinary VOCs in mice with amygdala-dependent anxiety-depression and epilepsy. The hypothesis that altered urinary VOC profiles were derived from specific metabolic cascades could lead to the development of novel medications. Particularly, VOC biomarkers in urine samples could represent a simple and safe tool for primary screening by objective tests before using a questionnaire to assess the presence of major depression and the frailty status of older adults.

## Supplementary Information

Below is the link to the electronic supplementary material.

**Additional file 1.** Subjects’ medications consumed, sex, and age.

**Additional file 2.** Human urinary volatile organic compounds detected by gas chromatography and mass spectrometry using solid-phase micro-extraction and an HR20M column.

**Additional file 3.** Gas chromatography–mass spectrometry a and H-NMR diagrams (**b** and **c**) of texanol (1isobutyrate) and texanol isomer (3 isobutyrate).

**Additional file 4.** Urinary biomarkers with high predictive power by receiver operating characteristic curve analysis.

**Additional file 5.** Pearson’s correlation between the values of texanol (1-isobutyrate) and the texanol isomer (3-isobutyrate).

**Additional file 6.** Pearson’s bivariate correlations between combined volatile organic compound indices and cohort scores.

**Additional file 7.** Frequency of diet, alcohol and tobacco consumption, sleep outcomes, and subjects’ scores.

**Additional file 8.** Pearson’s bivariate correlations and single linear regression formula between combined volatile organic compound indices and cohort scores.

**Additional file 9.** Correlations between cohort assessments and combined three- or two-volatile organic compound indices.

## Data Availability

The data generated and analyzed in this paper are available from the corresponding author upon reasonable request.

## Code availability

The NIST 14 standard reference database (NIST/EPA/NIH mass spectral library). The XCMS software [39], package version 1.3.2 (http://masspec.scripps.edu) in R version 3.2.3 (http://cran.r-project.org/).

The GC–MS Solution software (ver. 4.45; Shimadzu).
