# Supplementary material for: A novel set of volatile urinary biomarkers for late-life major depressive and anxiety disorders upon the progression of frailty: a pilot study
Source: Discov Ment Health. 2022 Oct 27;2(1):20. doi: 10.1007/s44192-022-00023-0 (PMC10501039; doi:10.1007/s44192-022-00023-0)
Supplement: Supplementary file 1 — Additional file 1. Subjects’ medications consumed, sex, and age. [file 44192_2022_23_MOESM1_ESM.docx]

**Subjects’ medications consumed, sex, and age**

| Subject No. | Sex | Age | MDD and/or agoraphobia | Medication |
| --- | --- | --- | --- | --- |
| 1c | M | 78 | No | Bifidobacteria; Lansoprazole; MgO; Mosapride citrate hydrate; Pantethine |
| 2c | M | 73 | No | n.d. |
| 3c | M | 70 | No | L-Carbocisteine |
| 4c | F | 77 | No | No drug |
| 5c^#^ | F | 73 | No | n.d. |
| 6c | M | 85 | No | Olmesartan Medoxomil; Sennoside; Zolpidem tartrate |
| 7c | M | 79 | No | Allopurinol; Azelnidipine; Fluticasone furoate; Levothyroxine sodium hydrate; Olmesartan medoxomil; Propranolol hydrochloride; Sennoside; Thiamazole |
| 8c | F | 87 | No | n.d. |
| 9c | M | 75 | No | Ethyl icosapentate; Flavoxate hydrochloride; Tamsulosin hydrochloride |
| 10d | M | 84 | MDD | Dutasteride; Febuxostat; Naftopidil; Sennoside |
| 11da^#^ | M | 73 | MDD and Agoraphobia | Clotiazepam; Istradefyline; Levodopa; MgO; Mianserin hydrochloride; Sennoside |
| 12da | F | 74 | MDD and Agoraphobia | Bifidobacteria; Ethyl loflazepate; Famotidine; Lorazepam; MgO; Pravastatin sodium |
| 13d | F | 87 | MDD | Alfacalcidol, Amlodipine Besilate; Alprazolam; Lansoprazole; Menatetrenone; Methylcobalamin; MgO; Teprenone |
| 14a^#^ | M | 78 | Agoraphobia | Ambroxol hydrochloride; Cetirizine hydrochloride; Clarithromycin; L-Carbocisteine; Fluticasone furoate; Montelukast sodium; Naftopidil; Suplatast tosilate; Theophylline; Vilanterol trifenatate |
| 15d | F | 77 | MDD | Amlodipine Besilate; Etizolam; Fluvastaitin sodium |
| 16d | M | 79 | MDD | ATP-2Na; Azelnidipine; Clopidogrel sulfate; Ethyl icosapentate; Lansoprazole; Methylcobalamin; Mexiletine hydrochloride (JAN) carvedilol; Olmesartan Medoxomil; Pravastatin sodium |
| 17d | M | 77 | MDD | No drug |
| 18d | M | 67 | MDD | No drug |

^#^According to the personal statement, subject No. 5c had asthma. Medications suggested that subjects No.14a and No. 11da had asthma and Parkinson's disease, respectively.

n.d. not determined.
