# Supplementary material for: A novel set of volatile urinary biomarkers for late-life major depressive and anxiety disorders upon the progression of frailty: a pilot study
Source: Discov Ment Health. 2022 Oct 27;2(1):20. doi: 10.1007/s44192-022-00023-0 (PMC10501039; doi:10.1007/s44192-022-00023-0)
Supplement: Supplementary file 2 — Additional file 2. Human urinary volatile organic compounds detected by gas chromatography and mass spectrometry using solid-phase micro-extraction and an HR20M column. [file 44192_2022_23_MOESM2_ESM.docx]

**Human urinary volatile organic compounds detected by gas chromatography and mass spectrometry using solid-phase micro-extraction and an HR20M column**

| No. | RT | SI | VOCs | Formula | CAS | M.W. |
| --- | --- | --- | --- | --- | --- | --- |
| 1 | 4.426 | 91 | Methyl Isobutyl Ketone | C6H12O | 108-10-1 | 100 |
| 2 | 4.975 | 98 | Trichloromethane | CHCl3 | 67-66-3 | 118 |
| 3 | 5.475 | 98 | Toluene | C7H8 | 108-88-3 | 92 |
| 4 | 5.638 | 96 | 2,3-Hexanedione | C6H10O2 | 3848-24-6 | 114 |
| 5 | 6.527 | 86 | 2,3-Pentanedione | C5H8O2 | 600-14-6 | 100 |
| 6 | 6.559 | 94 | Dimethylamine | C2H7N | 124-40-3 | 45 |
| 7 | 6.732 | 87 | Disulfide, dimethyl | C2H6S2 | 624-92-0 | 94 |
| 8 | 6.964 | 85 | Tetradecane | C14H30 | 629-59-4 | 198 |
| 9 | 6.974 | 85 | 2,3,3-Trimethyloctane | C11H24 | 62016-30-2 | 156 |
| 10 | 6.976 | 86 | Decane, 2,6,8-trimethyl- | C13H28 | 62108-26-3 | 184 |
| 11 | 6.977 | 90 | Dodecane | C12H26 | 112-40-3 | 170 |
| 12 | 6.994 | 86 | Heptane, 2,2,3,3,5,6,6-heptamethyl- | C14H30 | 7225-67-4 | 198 |
| 13 | 7.002 | 86 | Undecane | C11H24 | 1120-21-4 | 156 |
| 14 | 7.003 | 85 | Tridecane | C13H28 | 629-50-5 | 184 |
| 15 | 7.059 | 85 | Oxalic acid, isobutyl nonyl ester | C15H28O4 | 0-00-0 ^#^ | 272 |
| 16 | 7.411 | 90 | Hexanal | C6H12O | 66-25-1 | 100 |
| 17 | 9.867 | 98 | 4-Heptanone | C7H14O | 123-19-3 | 114 |
| 18 | 10.56 | 85 | o-Xylene | C8H10 | 95-47-6 | 106 |
| 19 | 11.929 | 92 | Bicyclo[3.1.0]hex-2-ene, 2-methyl-5-(1-methylethyl)- | C10H16 | 2867-05-2 | 136 |
| 20 | 12.771 | 92 | (+)-2-Carene | C10H16 | 554-61-0 | 136 |
| 21 | 13.38 | 92 | 2-Heptanone | C7H14O | 110-43-0 | 114 |
| 22 | 13.816 | 87 | Cyclohexanol, 1-methyl-4-(1-methylethenyl)-, acetate | C12H20O2 | 10198-23-9 | 196 |
| 23 | 13.819 | 89 | D-Limonene | C10H16 | 5989-27-5 | 136 |
| 24 | 14.156 | 95 | Bicyclo[3.1.0]hexane, 4-methylene-1-(1-methylethyl)- | C10H16 | 3387-41-5 | 136 |
| 25 | 14.224 | 91 | Eucalyptol | C10H18O | 470-82-6 | 154 |
| 26 | 14.378 | 85 | Cyclopentanone, 3-methyl- | C6H10O | 1757-42-2 | 98 |
| 27 | 14.794 | 85 | 6,8-Dioxabicyclo[3.2.1]octane, 1,5-dimethyl-, (1S)- | C8H14O2 | 28401-39-0 | 142 |
| 28 | 15.92 | 88 | Nonane, 5-(2-methylpropyl)- | C13H28 | 62185-53-9 | 184 |
| 29 | 15.976 | 92 | 2-acetyl-2*H*-tetrazole* | C3H4N4O | 51410-11-8 | 112 |
| 30 | 16.012 | 92 | 1-Nitroacetone | C3H5NO3 | 10230-68-9 | 103 |
| 31 | 16.037 | 93 | .gamma.-Terpinene | C10H16 | 99-85-4 | 136 |
| 32 | 16.051 | 91 | Acetonitrile, (dimethylamino)- | C4H8N2 | 926-64-7 | 84 |
| 33 | 16.071 | 89 | Bromoacetone | C3H5BrO | 598-31-2 | 136 |
| 34 | 16.124 | 88 | Methyl glyoxal | C3H4O2 | 78-98-8 | 72 |
| 35 | 16.139 | 90 | Acetic acid, anhydride | C4H6O3 | 108-24-7 | 102 |
| 36 | 16.169 | 96 | Isocyanic acid | CHNO | 75-13-8 | 43 |
| 37 | 16.599 | 87 | Pyrrolidine, 3-methyl- | C5H11N | 34375-89-8 | 85 |
| 38 | 17.035 | 87 | o-Cymene | C10H14 | 527-84-4 | 134 |
| 39 | 17.288 | 87 | 4-Hexen-3-one | C6H10O | 2497-21-4 | 98 |
| 40 | 17.537 | 86 | Cyclohexanone | C6H10O | 108-94-1 | 98 |
| 41 | 17.657 | 93 | 2-Butanone, 3-hydroxy- | C4H8O2 | 513-86-0 | 88 |
| 42 | 18.18 | 95 | 2-Propanone, 1-hydroxy- | C3H6O2 | 116-09-6 | 74 |
| 43 | 18.243 | 85 | Methyl 3-oxobutanoate | C5H8O3 | 105-45-3 | 116 |
| 44 | 19.071 | 93 | 3-Ethylcyclopentanone | C7H12O | 10264-55-8 | 112 |
| 45 | 19.466 | 90 | 2,3-Octanedione* | C8H14O2 | 585-25-1 | 142 |
| 46 | 19.654 | 85 | Propanal, 2-hydroxy-2-methyl- | C4H8O2 | 20818-81-9 | 88 |
| 47 | 19.716 | 85 | Hydroperoxide, 1,1-dimethylethyl | C4H10O2 | 75-91-2 | 90 |
| 48 | 20.282 | 88 | 3-Hexanol, 2-methyl- | C7H16O | 617-29-8 | 116 |
| 49 | 20.5 | 96 | Allyl Isothiocyanate* | C4H5NS | 57-06-7 | 99 |
| 50 | 21.366 | 94 | Bicyclo[3.1.0]hex-2-ene, 4-methylene-1-(1-methylethyl)- | C10H14 | 36262-09-6 | 134 |
| 51 | 21.571 | 94 | Nonanal | C9H18O | 124-19-6 | 142 |
| 52 | 22.53 | 92 | Benzene, 1,3-bis(1,1-dimethylethyl)-* | C14H22 | 1014-60-4 | 190 |
| 53 | 22.963 | 91 | trans-Linalool oxide (furanoid) | C10H18O2 | 34995-77-2 | 170 |
| 54 | 23.261 | 98 | Acetic acid* | C2H4O2 | 64-19-7 | 60 |
| 55 | 23.376 | 96 | 1-Butene, 4-isothiocyanate* | C5H7NS | 3386-97-8 | 113 |
| 56 | 23.579 | 88 | 5-isopropyl-2-methylbicyclo [3.1.0]hexan-2-ol | C10H18O | 17699-16-0 | 154 |
| 57 | 24.427 | 98 | 2-Ethyl-1-hexanol | C8H18O | 104-76-7 | 130 |
| 58 | 24.473 | 88 | 1,3-Benzodioxole, 5-propyl- | C10H12O2 | 94-58-6 | 164 |
| 59 | 24.586 | 93 | Hexane, 1-nitro- | C6H13NO2 | 646-14-0 | 131 |
| 60 | 24.704 | 93 | (+)-2-Bornanone | C10H16O | 464-49-3 | 152 |
| 61 | 24.732 | 92 | Camphor | C10H16O | 76-22-2 | 152 |
| 62 | 24.974 | 95 | (1H)-pyrrole | C4H5N | 109-97-7 | 67 |
| 63 | 25.921 | 95 | Linalool | C10H18O | 78-70-6 | 154 |
| 64 | 26.009 | 98 | Dimethyl Sulfoxide* | C2H6OS | 67-68-5 | 78 |
| 65 | 26.193 | 95 | 4-Isopropyl-1-methylcyclohex-2-enol | C10H18O | 619-62-5 | 154 |
| 66 | 26.38 | 95 | Propanoic acid, 2-methyl- | C4H8O2 | 79-31-2 | 88 |
| 67 | 26.628 | 97 | Propanoic acid, 2,2-dimethyl- | C5H10O2 | 75-98-9 | 102 |
| 68 | 27 | 92 | 2,3-Butanediol | C4H10O2 | 513-85-9 | 90 |
| 69 | 27.182 | 95 | 1-Isopropyl-4-methyl-3-cyclohexan-1-ol | C10H18O | 562-74-3 | 154 |
| 70 | 27.187 | 88 | 2(3H)-Furanone, dihydro-5-methyl- | C5H8O2 | 108-29-2 | 100 |
| 71 | 27.648 | 89 | 2(3H)-Furanone, dihydro- | C4H6O2 | 96-48-0 | 86 |
| 72 | 27.874 | 95 | Butanoic acid | C4H8O2 | 107-92-6 | 88 |
| 73 | 28.173 | 98 | Menthol (Cyclohexanol, 5-methyl-2-(1-methylethyl)-, (1.alpha.,2.beta., 5.alpha.)-(.+/-.)-)* | C10H20O | 15356-70-4 | 156 |
| 74 | 28.233 | 89 | Levomenthol | C10H20O | 2216-51-5 | 156 |
| 75 | 28.33 | 85 | methyl vinyl sulfide | C3H6S | 1822-74-8 | 74 |
| 76 | 28.517 | 89 | cis-Verbenol | C10H16O | 1845-30-3 | 152 |
| 77 | 28.616 | 87 | (1R,4R,4aS,8aR)-4,7-Dimethyl-1-(prop-1-en-2-yl)-1,2,3,4,4a,5,6,8a-octahydronaphthalene | C15H24 | 92692-39-2 | 204 |
| 78 | 28.904 | 95 | Butanoic acid, 3-methyl- | C5H10O2 | 503-74-2 | 102 |
| 79 | 29.043 | 94 | Bicyclo[3.1.1]hept-3-en-2-ol, 4,6,6-trimethyl-, [1S-(1.alpha.,2.beta.,5.alpha.)]- | C10H16O | 18881-04-4 | 152 |
| 80 | 29.198 | 85 | 1-(1,3-benzodioxol-5-yl)-N-(1,3-benzodioxol-4-ylmethyl)-N-methylpropan-2-amine | C19H21NO4 | 0-00-0 ^#^ | 327 |
| 81 | 29.229 | 86 | 2-Methoxy-4-methyl-1-pentylbenzene | C13H20O | 0-00-0 ^#^ | 192 |
| 82 | 29.413 | 91 | Propane, 1-(methylsulfinyl)- | C4H10OS | 14094-08-7 | 106 |
| 83 | 29.446 | 96 | 1-Methyl-4-(6-methylhept-5-en-2-yl)cyclohexa-1,3-diene | C15H24 | 451-55-8 | 204 |
| 84 | 29.505 | 87 | 1,6-Octadien-3-ol, 3,7-dimethyl-, propanoate | C13H22O2 | 144-39-8 | 210 |
| 85 | 29.547 | 93 | Benzaldehyde, 2-chloro- | C7H5ClO | 89-98-5 | 140 |
| 86 | 30.158 | 95 | (1S,5S)-2-Methyl-5-((R)-6-methylhept-5-en-2-yl)bicyclo[3.1.0]hex-2-ene | C15H24 | 159407-35-9 | 204 |
| 87 | 30.168 | 94 | 2-Cyclohexen-1-one, 3-methyl-6-(1-methylethyl)- | C10H16O | 89-81-6 | 152 |
| 88 | 30.193 | 90 | 2,4-Cyclohexadiene-1-methanol, .alpha.,.alpha.,4-trimethyl- | C10H16O | 1686-20-0 | 152 |
| 89 | 30.197 | 90 | 1,3-Cyclohexadiene, 5-(1,5-dimethyl-4-hexenyl)-2-methyl-, [S-(R*,S*)]- | C15H24 | 495-60-3 | 204 |
| 90 | 30.289 | 96 | (-)-Carvone* | C10H14O | 6485-40-1 | 150 |
| 91 | 30.476 | 97 | Pentanoic acid | C5H10O2 | 109-52-4 | 102 |
| 92 | 30.617 | 94 | (R)-1-Methyl-4-(6-methylhept-5-en-2-yl)cyclohexa-1,4-diene | C15H24 | 28976-67-2 | 204 |
| 93 | 30.649 | 88 | 2-Cyclohexen-1-ol, 3-methyl-6-(1-methylethyl)-, trans- | C10H18O | 16721-39-4 | 154 |
| 94 | 30.782 | 98 | Acetamide | C2H5NO | 60-35-5 | 59 |
| 95 | 31.255 | 93 | Methyl salicylate | C8H8O3 | 119-36-8 | 152 |
| 96 | 31.262 | 91 | beta-Sesquiphellandrene | C15H24 | 20307-83-9 | 204 |
| 97 | 31.716 | 94 | 2-Piperidinone, 1-methyl- | C6H11NO | 931-20-4 | 113 |
| 98 | 32.077 | 98 | Benzaldehyde, 2,4-dimethyl- | C9H10O | 15764-16-6 | 134 |
| 99 | 32.545 | 90 | 2-Propanol, 1,1'-oxybis- | C6H14O3 | 110-98-5 | 134 |
| 100 | 32.61 | 85 | (3R,4aS,8aS)-8a-Methyl-5-methylene-3-(prop-1-en-2-yl)-1,2,3,4,4a,5,6,8a-octahydronaphthalene | C15H22 | 212394-95-1 | 202 |
| 101 | 32.727 | 95 | Formamide, N,N-dimethyl- | C3H7NO | 68-12-2 | 73 |
| 102 | 32.89 | 99 | Hexanoic acid** | C6H12O2 | 142-62-1 | 116 |
| 103 | 33.102 | 85 | Ethanol, 2-(2-butoxyethoxy)-, acetate | C10H20O4 | 124-17-4 | 204 |
| 104 | 33.125 | 91 | Pyridine, 3-(1-methyl-2-pyrrolidinyl)-, (S)- | C10H14N2 | 54-11-5 | 162 |
| 105 | 33.463 | 97 | 2,2,4-trimethyl-1,3-pentanediol 1-monoisobutyrate (texanol)** | C12H24O3 | 25265-77-4 | 216 |
| 106 | 33.654 | 92 | 1-Propanol, 2-(2-hydroxypropoxy)- | C6H14O3 | 106-62-7 | 134 |
| 107 | 33.699 | 92 | 2,2,4-trimethyl-1,3-pentanediol 3-monoisobutyrate (texanol isomer) ** | C12H24O3 |  | 216 |
| 108 | 33.733 | 93 | Propanoic acid, 2-methyl-, 2,2-dimethyl-1-(2-hydroxy-1-methylethyl)propyl ester | C12H24O3 | 74367-33-2 | 216 |
| 109 | 33.836 | 98 | Dimethyl sulfone* | C2H6O2S | 67-71-0 | 94 |
| 110 | 34.17 | 92 | Phenol, 2,5-dimethyl- | C8H10O | 95-87-4 | 122 |
| 111 | 34.263 | 90 | 2-Cyclopentene, 1-hydroxymethyl-1,2,3-trimethyl- | C9H16O | 0-00-0 ^#^ | 140 |
| 112 | 34.471 | 90 | 1,4-Butanediol | C4H10O2 | 110-63-4 | 90 |
| 113 | 35.05 | 87 | Benzothiazole* | C7H5NS | 95-16-9 | 135 |
| 114 | 35.158 | 93 | Hexanoic acid, 2-ethyl- | C8H16O2 | 149-57-5 | 144 |
| 115 | 35.283 | 87 | Propanoic acid, decyl ester | C13H26O2 | 5454-19-3 | 214 |
| 116 | 35.463 | 90 | 2-(Methylsulphonyl)propane | C4H10O2S | 4853-74-1 | 122 |
| 117 | 35.61 | 99 | 1-Dodecanol | C12H26O | 112-53-8 | 186 |
| 118 | 35.612 | 96 | Cyclododecane | C12H24 | 294-62-2 | 168 |
| 119 | 35.733 | 90 | Propane, 1-isothiocyanato-3-(methylthio)- | C5H9NS2 | 505-79-3 | 147 |
| 120 | 35.809 | 90 | Oxalic acid, diallyl ester | C8H10O4 | 0-00-0 ^#^ | 170 |
| 121 | 35.976 | 85 | Isolongifolene, 4,5,9,10-dehydro- | C15H20 | 156747-45-4 | 200 |
| 122 | 36.121 | 97 | Phenol* | C6H6O | 108-95-2 | 94 |
| 123 | 36.157 | 90 | 2-Propenoic acid, tridecyl ester | C16H30O2 | 3076-04-8 | 254 |
| 124 | 36.27 | 88 | 2-[(Acetyl)methoxy]-1,3-dimethylbenzene | C11H14O2 | 0-00-0 ^#^ | 178 |
| 125 | 36.914 | 87 | Cyclohexane, ethenyl- | C8H14 | 695-12-5 | 110 |
| 126 | 37.043 | 89 | Urea, N,N-dimethyl- | C3H8N2O | 598-94-7 | 88 |
| 127 | 37.351 | 98 | Octanoic acid | C8H16O2 | 124-07-2 | 144 |
| 128 | 37.627 | 97 | p-Cresol* | C7H8O | 106-44-5 | 108 |
| 129 | 37.691 | 96 | (.+/-.)-Dihydro-ar-turmerone | C15H22O | 30666-87-6 | 218 |
| 130 | 38.052 | 93 | Benzene, (isothiocyanatomethyl)- | C8H7NS | 622-78-6 | 149 |
| 131 | 38.214 | 98 | 2-Piperidinone** | C5H9NO | 675-20-7 | 99 |
| 132 | 38.415 | 90 | (1R,4R)-1-methyl-4-(6-Methylhept-5-en-2-yl)cyclohex-2-enol | C15H26O | 58334-55-7 | 222 |
| 133 | 38.602 | 92 | Erucin | C6H11NS2 | 4430-36-8 | 161 |
| 134 | 38.741 | 90 | 2-(6,10-dimethylspiro[4.5]dec-6-en-2-yl)-2-propanol | C15H26O | 1460-73-7 | 222 |
| 135 | 38.964 | 87 | 2-(4a,8-Dimethyl-2,3,4,5,6,8a-hexahydro-1H-naphthalen-2-yl)propan-2-ol | C15H26O | 0-00-0 ^#^ | 222 |
| 136 | 39.164 | 89 | 3-Cyclohexen-1-ol, 1-(1,5-dimethyl-4-hexenyl)-4-methyl- | C15H26O | 15352-77-9 | 222 |
| 137 | 39.175 | 88 | Formamide, N-phenyl- | C7H7NO | 103-70-8 | 121 |
| 138 | 39.179 | 90 | Benzaldehyde, 2-amino- | C7H7NO | 529-23-7 | 121 |
| 139 | 39.316 | 94 | 3-Allyl-6-methoxyphenol | C10H12O2 | 501-19-9 | 164 |
| 140 | 39.796 | 90 | 2-Methoxy-4-vinylphenol* | C9H10O2 | 7786-61-0 | 150 |
| 141 | 39.86 | 98 | 2,5-dichlorophenol* | C6H4Cl2O | 583-78-8 | 162 |
| 142 | 40.214 | 93 | 2-Methyl-6-(p-tolyl)hept-2-en-4-ol | C15H22O | 38142-57-3 | 218 |
| 143 | 40.308 | 97 | Phenethyl isothiocyanate* | C9H9NS | 2257-09-2 | 163 |
| 144 | 40.624 | 86 | Methyl anthranilate | C8H9NO2 | 134-20-3 | 151 |
| 145 | 41.035 | 91 | Berteroin | C7H13NS2 | 4430-42-6 | 175 |
| 146 | 41.044 | 94 | aR-Turmerone | C15H20O | 532-65-0 | 216 |
| 147 | 41.191 | 86 | Cyclodecane | C10H20 | 293-96-9 | 140 |
| 148 | 41.438 | 96 | Decanoic acid | C10H20O2 | 334-48-5 | 172 |
| 149 | 42.1 | 98 | Phenol, 2,4-bis(1,1-dimethylethyl)- | C14H22O | 96-76-4 | 206 |
| 150 | 44.119 | 93 | Indole | C8H7N | 120-72-9 | 117 |
| 151 | 44.47 | 93 | Benzoic acid | C7H6O2 | 65-85-0 | 122 |
| 152 | 44.476 | 85 | Benzoic acid, ammonium salt | C7H9NO2 | 1863-63-4 | 139 |
| 153 | 45.102 | 92 | Dodecanamide, N,N-bis(2-hydroxyethyl)- | C16H33NO3 | 120-40-1 | 287 |
| 154 | 45.119 | 94 | Dodecanoic acid | C12H24O2 | 143-07-7 | 200 |
| 155 | 46.032 | 89 | 1,2-Benzenedicarboxylic acid, bis (2-methylpropyl) ester | C16H22O4 | 84-69-5 | 278 |
| 156 | 46.755 | 91 | Urea | CH4N2O | 57-13-6 | 60 |
| 157 | 52.024 | 86 | n-Hexadecanoic acid | C16H32O2 | 57-10-3 | 256 |

RT, retention time; SI, similarity index; M.W., molecular weight.

^#^Seven species of volatile organic compounds (VOCs) do not have a CAS No.

^*^Sixteen VOCs manually nominated from total ion current peaks are shown in Table 2.

^**^Four VOCs selected by XCMS using R commands [39] are shown in Table 2.
