# Supplementary material for: A novel set of volatile urinary biomarkers for late-life major depressive and anxiety disorders upon the progression of frailty: a pilot study
Source: Discov Ment Health. 2022 Oct 27;2(1):20. doi: 10.1007/s44192-022-00023-0 (PMC10501039; doi:10.1007/s44192-022-00023-0)
Supplement: Supplementary file 3 — Additional file 3. Gas chromatography–mass spectrometry a and H-NMR diagrams (b and c) of texanol (1isobutyrate) and texanol isomer (3 isobutyrate). [file 44192_2022_23_MOESM3_ESM.docx]

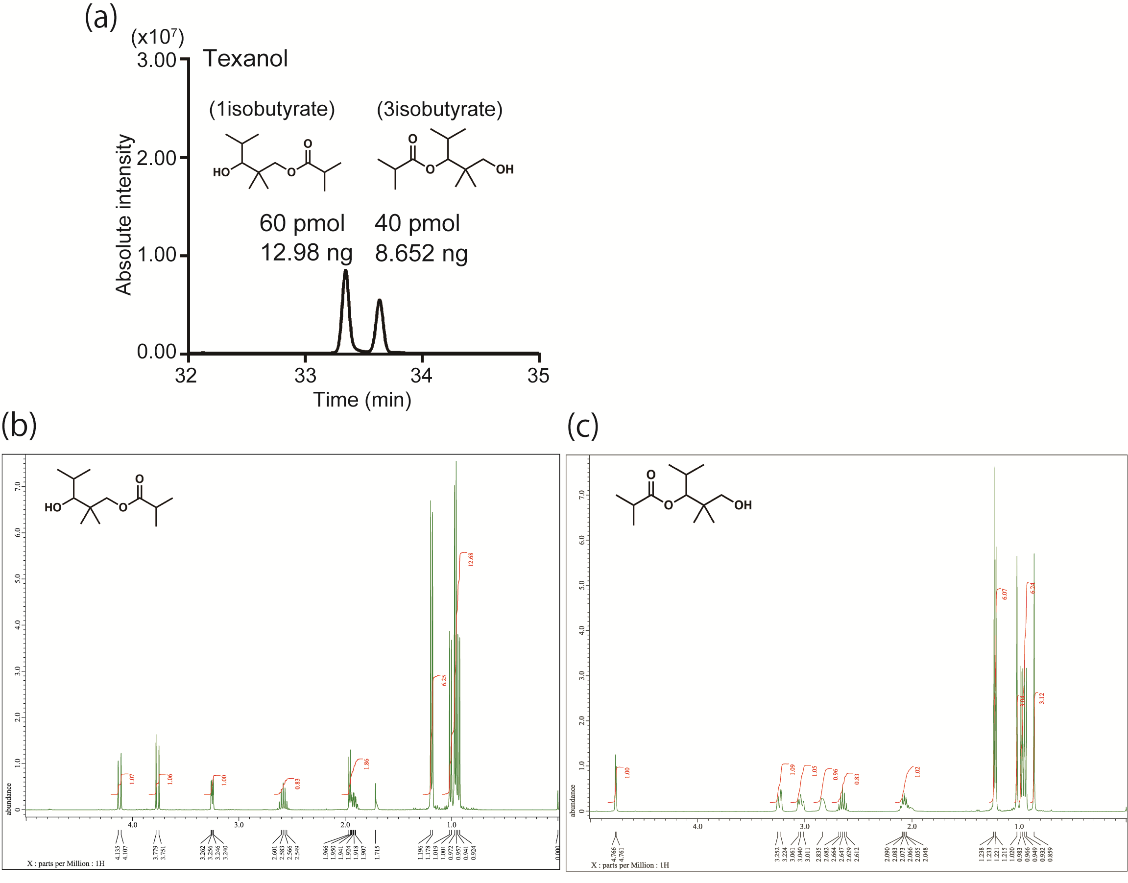


**Gas chromatography–mass spectrometry (a) and ^1^H-NMR diagrams (b and c) of texanol (1-isobutyrate) and texanol isomer (3-isobutyrate)**

Commercial 2,2,4-trimethyl-1,3-pentanediol monoisobutyrate (Texanol^Ⓡ^, catalog No. 40366, Alfa Aesar, Lancashire, UK) was composed of 60 and 40 pmol of texanol and texanol isomer, respectively (a).
