# Supplementary material for: A novel set of volatile urinary biomarkers for late-life major depressive and anxiety disorders upon the progression of frailty: a pilot study
Source: Discov Ment Health. 2022 Oct 27;2(1):20. doi: 10.1007/s44192-022-00023-0 (PMC10501039; doi:10.1007/s44192-022-00023-0)
Supplement: Supplementary file 4 — Additional file 4. Urinary biomarkers with high predictive power by receiver operating characteristic curve analysis. [file 44192_2022_23_MOESM4_ESM.docx]

**Urinary biomarkers with high predictive power by receiver operating characteristic curve analysis**

| NO^a)^ | Compound | Chemical class | Cut off | Sensitivity | Specificity | Accuracy | AUC | *p*-value | 95%  confidence interval | <http://www.hmdb.ca> ^$^ |
| --- | --- | --- | --- | --- | --- | --- | --- | --- | --- | --- |
| 1 | 2-acetyl-2*H*-tetrazole | ketone / tetrazole | >831358 | 0.8889 | 0.6667 | 0.7778 | 0.7407 | 0.0851 | 0.4892 to 0.9923 | n.d. |
| 10 | Dimethyl_sulfone | Organosulfur / hydrocarbone derivative | >701536 | 0.8889 | 0.5556 | 0.7222 | 0.7531 | 0.0703 | 0.5248 to 0.9813 | HMDB0004983 |
| 16 | Phenethyl isothiocyanate | Isothicyanate / aromatic | >22842 | 1.0000 | 0.6667 | 0.8333 | 0.8642 | 0.0092 | 0.6937 to 1.0350 | HMDB0038445 |
| 17 | Hexanoic_acid | Medium chain fatty acids | <333497 | 0.7778 | 0.8889 | 0.8333 | 0.8519 | 0.0118 | 0.6700 to 1.0340 | HMDB0000535 |
| 18 | Texanol | Hydrocarbone | >318658 | 1.0000 | 0.8889 | 0.9444 | 0.9877 | 0.0005 | 0.9489 to 1.0260 | HMDB0059777 |
| 19 | Texanol isomer | Hydrocarbone | >372470 | 0.8889 | 0.7778 | 0.8333 | 0.8889 | 0.0054 | 0.7393 to 1.0390 | n.d. |
|  | Combined index^#^ six VOCs | n.d. | >0.4119 | 1.0000 | 1.0000 | 1.0000 | 1.0000 | 0.0003 | 1.0000 to 1.0000 | n.d. |
|  | Combined index^#^ five VOCs (No.10,16〜19) | n.d. | >0.3475 | 1.0000 | 0.8889 | 0.9444 | 0.9877 | 0.0005 | 0.9489 to 1.0260 | n.d. |
|  | Combined index^#^ three VOCs (No.10, 16, 17) | n.d. | >0.3382 | 1.0000 | 0.6667 | 0.8333 | 0.9136 | 0.0031 | 0.7839 to 1.0430 | n.d. |
|  | Combined index^#^ two VOCs (No. 18, 19) | n.d. | >0.4379 | 1.0000 | 0.8889 | 0.9444 | 0.9877 | 0.0005 | 0.9489 to 1.0260 | n.d. |

Receiver operating characteristic (ROC) curve analyses were performed on the absolute values of six volatile organic compounds (VOCs) screened in Table 2.

^a)^ VOC numbers refer to Table 2: 2-acetyl-2*H*-tetrazole and dimethyl sulfone showed a fair ROC curve (area under the curve [AUC] <0.8); phenethyl isothiocyanate, hexanoic acid, and texanol isomer showed good ROC curves (0.8<AUC<0.9); texanol showed an excellent curve (AUC >0.9).

^#^Linear regression was computed based on the absolute values of 2–6 VOCs in each participant. The unstandardized predicted values (PRE-1) extracted from 18 participants, including the negative and positive values, were then applied to analyze the combined indices of ROC curves. The combined indices of the six VOCs showed a complete AUC (AUC = 1). All combined indices of five VOCs, including dimethyl sulfone (10), phenethyl isothiocyanate (16), hexanoic acid (17), texanol (18), and texanol isomer (19); three VOCs, including dimethyl sulfone, phenethyl isothiocyanate, and hexanoic acid; and two VOCs, including texanol and texanol isomer, showed excellent AUCs (>0.9).
^$^Compounds have been detected in human specimens, such as urine, feces, and blood (<http://www.hmdb.ca>).

n.d. “not described.”
