# Supplementary material for: A novel set of volatile urinary biomarkers for late-life major depressive and anxiety disorders upon the progression of frailty: a pilot study
Source: Discov Ment Health. 2022 Oct 27;2(1):20. doi: 10.1007/s44192-022-00023-0 (PMC10501039; doi:10.1007/s44192-022-00023-0)
Supplement: Supplementary file 5 — Additional file 5. Pearson’s correlation between the values of texanol (1-isobutyrate) and the texanol isomer (3-isobutyrate). [file 44192_2022_23_MOESM5_ESM.docx]

**
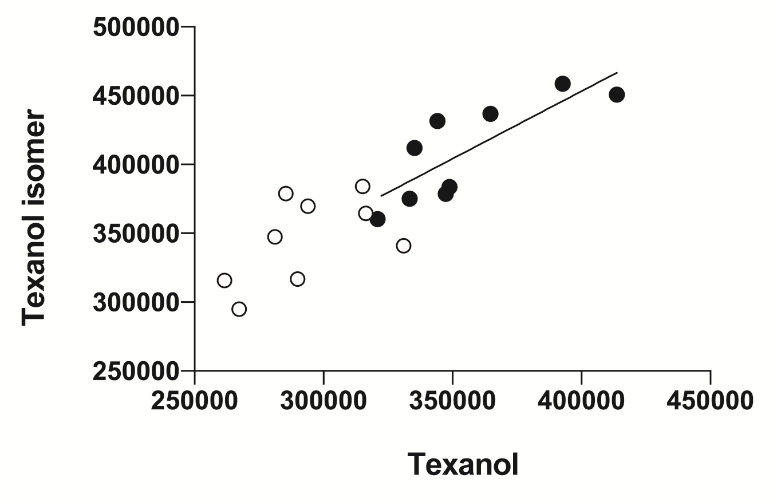
**

**Pearson's correlation between the values of texanol (1-isobutyrate) and the texanol isomer (3-isobutyrate)**

The formulae and the corresponding *r*- and *p*-values were analyzed using Prism 8. Formulae, *r- and p*-values: Y=60804+0.981*X, *r*=0.809, *p*=0.008 in major depressive disorder (MDD) and/or anxiety positive persons (black circles and line); Y=129602+0.737*X, *r*=0.548, *p*=0.127 in negative persons (white circles).
