# Supplementary material for: A novel set of volatile urinary biomarkers for late-life major depressive and anxiety disorders upon the progression of frailty: a pilot study
Source: Discov Ment Health. 2022 Oct 27;2(1):20. doi: 10.1007/s44192-022-00023-0 (PMC10501039; doi:10.1007/s44192-022-00023-0)
Supplement: Supplementary file 6 — Additional file 6. Pearson’s bivariate correlations between combined volatile organic compound indices and cohort scores. [file 44192_2022_23_MOESM6_ESM.docx]

**Pearson’s bivariate correlations between combined volatile organic compound indices and cohort scores**

| Cohort items^a)^ | Combined index of VOCs^b)^ |  | Six VOCs (No. 1〜6) | Five VOCs (No. 2〜6) | Three VOCs (No. 2, 3, 6) | Two VOCs (No. 4, 5) | Urinary creatinine |
| --- | --- | --- | --- | --- | --- | --- | --- |
| Activity indices for the classification of frailty |  |  |  |  |  |  |  |
|  | Kihon CL score | Pearson-*r* ^c)^ | 0.5651 | 0.5546 | 0.3319 | 0.6189 | 0.04180 |
|  |  | 95% confidence interval | 0.1334 to 0.8165 | 0.1184 to 0.8114 | -0.1598 to 0.6916 | 0.2138 to 0.8424 | -0.4335 to 0.4989 |
|  |  | *p* value (two-tailed) | 0.0145* | 0.0169* | 0.1785 | 0.0062** | 0.8692 |
|  | TMIG-IC | Pearson- *r* | -0.1240 | -0.1235 | -0.09557 | -0.04854 | 0.2845 |
|  |  | 95% confidence interval | -0.5585 to 0.3640 | -0.5582 to 0.3644 | -0.5384 to 0.3886 | -0.5040 to 0.4280 | -0.2103 to 0.6633 |
|  |  | *p* value (two-tailed) | 0.6241 | 0.6255 | 0.7060 | 0.8483 | 0.2526 |
|  | JST-IC | Pearson- *r* | -0.1606 | -0.1783 | -0.1204 | -0.1455 | -0.02811 |
|  |  | 95% confidence interval | -0.5837 to 0.3311 | -0.5956 to 0.3148 | -0.5560 to 0.3671 | -0.5734 to 0.3448 | -0.4886 to 0.4446 |
|  |  | *p* value (two-tailed) | 0.5244 | 0.4790 | 0.6341 | 0.5645 | 0.9118 |
| Depression-related tests |  |  |  |  |  |  |  |
|  | DSKC | Pearson- *r* | 0.4469 | 0.4436 | 0.3939 | 0.2980 | -0.01055 |
|  |  | 95% confidence interval | -0.02524 to 0.7560 | -0.02940 to 0.7542 | -0.08945 to 0.7270 | -0.1962 to 0.6714 | -0.4751 to 0.4586 |
|  |  | *p* value (two-tailed) | 0.0630 | 0.0652 | 0.1058 | 0.2298 | 0.9669 |
|  | DSKC-self | Pearson- *r* | 0.7877 | 0.7818 | 0.6519 | 0.6733 | -0.07083 |
|  |  | 95% confidence interval | 0.5074 to 0.9172 | 0.4959 to 0.9148 | 0.2660 to 0.8577 | 0.3010 to 0.8675 | -0.5205 to 0.4096 |
|  |  | *p* value (two-tailed) | 0.0001*** | 0.0001*** | 0.0034** | 0.0022** | 0.7800 |
|  | SDS | Pearson- *r* | 0.4548 | 0.4701 | 0.5412 | 0.2653 | -0.1742 |
|  |  | 95% confidence interval | -0.01531 to 0.7603 | 0.004124 to 0.7683 | 0.09941 to 0.8047 | -0.2301 to 0.6515 | -0.5929 to 0.3186 |
|  |  | *p value (two-tailed)* | 0.0579 | 0.0490* | 0.0204* | 0.2873 | 0.4893 |
|  | GRID-HAMD (wo No. 14a)^d)^ | Pearson- *r* | 0.5612 | 0.6310 | 0.7292 | 0.1045 | 0.06408 |
|  |  | 95% confidence interval | -0.2373 to 0.9071 | -0.1326 to 0.9246 | 0.05037 to 0.9472 | -0.6479 to 0.7537 | -0.6709 to 0.7355 |
|  |  | *p* value (two-tailed) | 0.1478 | 0.0934 | 0.0401* | 0.8056 | 0.8802 |
| Biochemical assays |  |  |  |  |  |  |  |
|  | Plasma creatinine | Pearson- *r* | 0.04537 | 0.02213 | -0.1355 | 0.01703 | 0.3241 |
|  |  | 95% confidence interval | -0.4607 to 0.5292 | -0.4788 to 0.5122 | -0.5914 to 0.3862 | -0.4827 to 0.5084 | -0.2045 to 0.7063 |
|  |  | *p* value (two-tailed) | 0.8675 | 0.9352 | 0.6170 | 0.9501 | 0.2208 |
|  | Urinary creatinine | Pearson- *r* | 0.08728 | 0.1784 | 0.08088 | 0.1706 | 1.0000 |
|  |  | 95% confidence interval | -0.3957 to 0.5325 | -0.3147 to 0.5956 | -0.4011 to 0.5278 | -0.3219 to 0.5904 | n.d. |
|  |  | *p* value (two-tailed) | 0.7306 | 0.4788 | 0.7497 | 0.4986 | n.d. |
|  | Urinary specific gravity | Pearson- *r* | 0.1108 | 0.2085 | 0.04245 | 0.2673 | 0.7081 |
|  |  | 95% confidence interval | -0.3755 to 0.5493 | -0.2862 to 0.6155 | -0.4330 to 0.4994 | -0.2281 to 0.6527 | 0.3604 to 0.8830 |
|  |  | *p* value (two-tailed) | 0.6615 | 0.4063 | 0.8672 | 0.2836 | 0.0010** |

Pearson’s bivariate correlations were computed to study the association between cohort assessment items ^a)^ (summarized in Table 1) and combined indices volatile organic compound (VOCs) ^b)^ (summarized in Online Resource 2) in the 18 negative and positive participants. ^c)^ Pearson's coefficient (*r*). ^d)^ The correlation of VOCs with the GRID-Hamilton Rating Scale for Depression (GRID-HAMD) was calculated using eight positive persons, after excluding one person (No. 14a) who exhibited symptoms of agoraphobia but not major depressive disorder, and nine negative persons.

Kihon CL, Kihon Checklist that consists of 20 items [14]; TMIG-IC, Tokyo Metropolitan Institute of Gerontology Index of Competence [31]; JST-IC, Japan Science and Technology Agency Index of Competence [15]; DSKC, Depression subdomain in the Kihon checklist [32]; SDS, Self-rating Depression Scale [33].
