# Supplementary material for: A novel set of volatile urinary biomarkers for late-life major depressive and anxiety disorders upon the progression of frailty: a pilot study
Source: Discov Ment Health. 2022 Oct 27;2(1):20. doi: 10.1007/s44192-022-00023-0 (PMC10501039; doi:10.1007/s44192-022-00023-0)
Supplement: Supplementary file 9 — Additional file 9. Correlations between cohort assessments and combined three- or two-volatile organic compound indices. [file 44192_2022_23_MOESM9_ESM.docx]

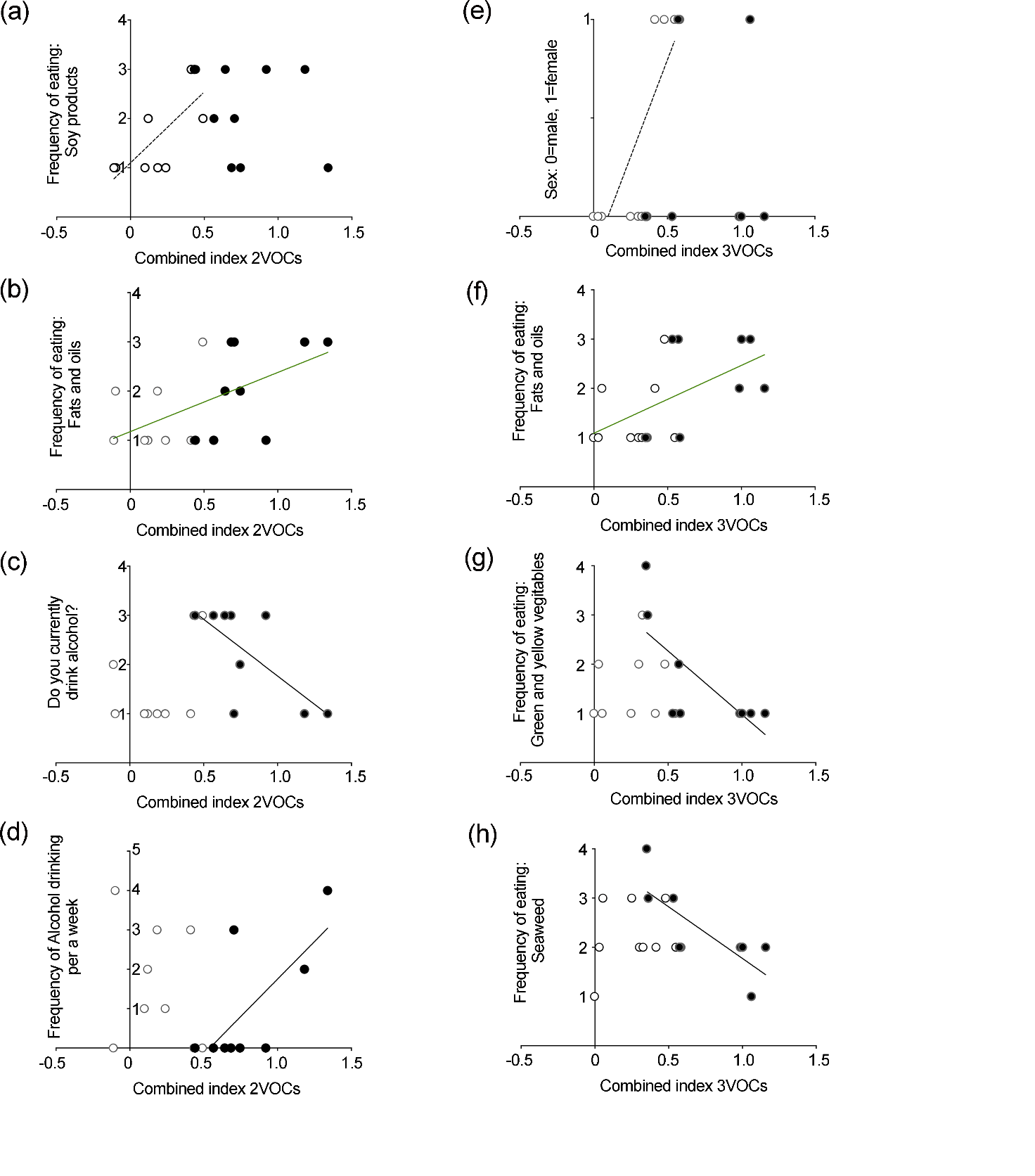


**Correlations between cohort assessments and combined three- or two-volatile organic compound indices**

Graphs showing any correlations are listed in Online Resource 8. The linear estimations showed significant Pearson’s correlations between cohort assessments and combined volatile organic compound (VOC) indices: frequency of eating soy products (a), fats and oils (b) and (f), green and yellow vegetables (g), and seaweed (h) (1: almost every day, 2: once every two days, 3: once or twice a week, 4: rarely eaten); Do you currently drink alcohol? (c) (1: I drink, 2: I have stopped drinking, 3: I have not drunk before); How many days a week do you drink? (d); Sex (e). Major depressive disorder and/or anxiety positive (black circles) and negative persons (white circles). Green line (all participants), black line (MDD/agoraphobia people), dotted line (control people). The formulae and the corresponding *r*-values and *p*-values were analyzed using Prism 8. Formulae, *r- and p*-values: Y=2.885*X+1.099, *r*=0.735, F(1, 7)=8.218, *p*=0.024 in control people (a); Y=1.202*X+1.177, *r*=0.549, F(1, 16)=6.886, *p*=0.018 in all participants (b); Y=2.305*X+4.074, *r*= -0.694, F(1, 7)=6.490, *p*=0.038 in MDD/agoraphobia people (c); Y=3.812*X+2.062, *r*=0.705, F(1, 7)=6.921, *p*=0.034 in MDD/agoraphobia people (d); Y=1.974*-0.192, *r*=0.796, F(1, 7)=12.077, *p*=0.010 in control people (e); Y=1.378*X+1.089, *r*=0.552, F(1, 16)=7.004, *p*=0.018 in all participants (f); Y=2.583*X+3.562, *r*= -0.728, F(1, 7)=7.911, *p*=0.026 in MDD/agoraphobia people (g); Y=2.096*X+3.871, *r*= -0.763, F(1, 7)=9.751, *p*=0.017 in MDD/agoraphobia people (h).
